# Syntaxin-1a and SNAP-25 expression level is increased in the blood samples of ischemic stroke patients

**DOI:** 10.1038/s41598-022-18719-2

**Published:** 2022-08-25

**Authors:** Pamela Cappelletti, Melania Filareti, Laura Masuelli, Roberto Bei, Kambiz Hassanzadeh, Massimo Corbo, Marco Feligioni

**Affiliations:** 1 Department of Neuro-Rehabilitation Sciences, Casa Cura Policlinico, Milan, Italy; 2 Department of Experimental Medicine, University of Rome “Sapienza”, Rome, Italy; 3 Department of Clinical Sciences and Translational Medicine, University of Rome “Tor Vergata”, Rome, Italy; 4 European Brain Research Institute (EBRI) Rita Levi Montalcini Foundation, Viale Regina Elena 295, 00161 Rome, Italy; 5 Present Address: Fondazione Pisana per la Scienza (FPS), Pisa, Italy

**Keywords:** Biochemistry, Neuroscience

## Abstract

The interest for the discovery of blood biomarkers for several neurological disorders, including Ischemic Stroke (IS), is growing and their identification in blood samples would be revolutionary allowing a fast and better pathology prediction or outcome and to collect information on patient recovery. The increased permeability of the blood–brain barrier, following a brain infarct, allows the detection of brain proteins in the blood flow. In this work, we analyzed the expression levels of two synaptic proteins Syntaxin (STX)-1a and Synaptosomal Associated Protein, 25 kDa (SNAP-25), in Peripheral Blood Mononuclear Cell (PBMC), serum and in Neuronal Derived Extracellular vesicles (NDEs) of IS patients, age and sex matched healthy control (HC) and younger HC (Y-HC). Interestingly, we identified STX-1a protein in the cytoplasm of PBMC and both STX-1a and SNAP-25 expression levels were significantly augmented in all IS patient’s blood fractions compared to control subjects. In addition, STX-1a blood levels correlated with the IS clinical scales National Institutes of Health Stroke Scale (NIH-SS) and the modified Barthel Index (BI). These results prompted us to speculate that STX-1a and SNAP-25 hematic fluctuations depict the brain damage after an ischemic attack and that their hematic detection could represent a novel and accessible IS biomarkers.

## Introduction

Ischemic Stroke is a severe pathology originating from a thrombotic or embolic event that reduces blood supply to the brain^1^. It represents the third leading cause of death in the industrialized countries (40–60% of IS patients die within 5 years after the ischemic event) and the second most common origin of dementia^2,3^. The current therapies and patient’s management are not sufficient to increase the lifetime expectancy after stroke^4,5^. One of the main obstacles encountered in ordinary therapies is certainly the heterogeneous nature of the pathology and the patient’s individual variability. Indeed, the possibility to tailor specific therapies for each patient is a medical unmet need^6^.

The brain tissue affected by stroke events presents two distinct areas of damage that can be identified as the ischemic core, in which the blood flow is completely absent and neuronal death occurs within a few minutes, and the penumbra, in which the blood flow is moderately reduced and the brain tissues, although functionally impaired, is still semi-viable^7^. Neurons located in the ischemic core region undergo death, mainly through necrosis mechanisms including the lack of ATP (Adenosine Tri-Phospahte), increased concentrations of ions and glutamate, and tissue acidosis^8^. By contrast, neurons in the ischemic penumbra undergo a similar fate if blood flow (and therefore, oxygen and glucose supply) is not restored within a short time. Neurons in the penumbra area die because of the strong activation of the apoptotic pathway driven by the augmentation of Ca2+ into cells that causes cells death for the excess of glutamate released^9^.

The over-flow of glutamate and its persistence in the synaptic cleft induces a cascade of biochemical events, known as ‘excitotoxicity’, which includes a prolonged activation of glutamate receptors and forms a vicious circle between elevated concentrations of intracellular Ca2+ ions and aberrant glutamate release, worsening at the end the effect of the ischemic event and leading to neuronal cells loss^10^.

The release of neurotransmitters, including glutamate, relies on a well-studied molecular mechanism which involves a group of proteins, implicated in the synaptic release of glutamate, that form a complex called SNAREs. Indeed, it is reported that the disruption of the SNARE complex formation induces the neurotransmitter release disorder especially by cleaving SNAP-25, a key SNARE complex protein^11–14^.

Another essential partner for the formation of SNARE complex is STX-1a, a protein anchored to the presynaptic membrane, which requires a switch from closed to open conformation to bind SNAP-25 and participate in the release of neurotransmitters^15^.

Few evidence highlighted the involvement of both STX-1a and SNAP-25 in IS, but still, further research is needed to understand their contribution to this pathology. Indeed, the protein level of STX-1a is markedly up-regulated in the cortex of a rat model of IS, probably suggesting the attempt of the brain to preserve the neuronal synaptic function after cerebral ischemia^16^. The correlation between SNAP-25 and IS is not clear: the mRNA (Messanger Ribonucleic Acid) levels of SNAP-25 has been found increased in the mossy fiber layer of gerbils up to 7 days after induced transient ischemia lasted for 5 min^17^ while, another study on rat models indicates that IS induced its protein reductions^18^.

Several proteins such as GFAP (Glial Fibrillary Acid Protein), S100β (S100 calcium-binding protein B), NSE (Neuron Specific Enolase), Lp-PLA 2 (Lipoprotein-associated Phospholipase A2), MMP-9 (Matrix Metalloproteinase-9), D-dimer and HSP70 (Heat Shock Protein 70)^19,20^, has been proposed as clinical biomarkers for IS, but none of them has reached successfully the clinical usage. Alterations of synaptic proteins levels, instead, have been proposed as pathological biomarkers for Alzheimer’s disease^21^, therefore, the detection of these proteins in human biological fluid samples has raised scientific interest.

Recently, the extracellular vesicles isolated from blood samples of patients have been indicated as a valuable biological sample to identify pathological biomarkers^22^. Extracellular vesicles, and in particular exosomes, which are endosome-derived small membrane vesicles (30–150 nm size) carry biological active molecules (such as genetic material, proteins and lipids), interact with neighboring cells to transmit their cargo from cells to cells, thus playing an essential role in intercellular communication^23,24^. Brain cells, including neurons, also release extracellular vesicles into the extracellular milieu to be then uptaken by neighboring cells or pass into the cerebrospinal fluid and blood^25,26^. Recent studies have reported that neuronal derived exosomes, found in the blood of patients, carry substances associated with neurological diseases^27^.

Interestingly, it has been demonstrated that STX-1a, known as presynaptic protein, is potentially expressed in human blood cells in which a wide number of syntaxins was found^28^. In addition, the STX-1a mRNA, but not the mRNA for STX-1b and SNAP-25, has been detected in human CD8 + T cells^29^. On the other hand, SNAP-25 has been assessed in human serum^30^ while its expression in PBMCs has never been detected^29,31^*.* More recently, both STX-1a and SNAP-25 have been measured in neuronal derived exosomes and proposed as potential biomarkers for neurodegenerative diseases^30,32^. However, the expression of these two proteins in blood samples of IS patients still has not been described.

Therefore, in this work, we showed the presence of both STX-1a and SNAP-25 in human blood fractions: serum, PBMCs and NDEs. We then analyze their expression levels in the blood components in a clinically characterized cohort of IS patients, HC and Y-HC subjects.

## Materials and methods

### Ethic approval

All methods were performed in accordance with the principles of the Declaration of Helsinki and the clinical protocol granted by the Ethics Committee of Milan: n° 285_2019 bis for sample collection and n° 729–2021 for the experimental study. All subjects have signed an informed consent.

### Study population

The blood samples were collected from 30 IS patients hospitalized in *Casa di Cura Privata del Policlinico* (CCPP) and selected based on the diagnosis, gender, age, and treatment. IS patients were evaluated based on the NIH-SS^33^ and BI^34^ scales used to quantify stroke severity and functional outcomes. 30 healthy IS-age-matched and 15 Y-HC controls were stratified by sex and age. IS and HC subjects were tested for pathology comorbidities through CIRS (Cumulative Illness Rating Scale) which measures the medical (such as hypertension, vascular, respiratory, hepatic, renal, endocrine-metabolic diseases) and psychiatric (such as dementia, depression, anxiety, agitation and psychosis) impairments in older adults^35^ and MMSE (Mini-Mental State Examination)^36^ for cognitive functions. We decided to use enrolled patients with minor or moderate stroke outcome (NIHSS up to 15) that could in the future have the better recovery so a biomarker could be more useful than in a patient with more severe pathology. All clinical scales were administered the same day of blood withdrawal. 59% of patients were under antiplatelet therapy, while 41% received both antiplatelet and anticoagulant therapy. Exclusion criteria were neurological comorbidity, psychiatric or oncological pathologies, and recent infections.

### Blood collection

Serum was collected in Clot activator tubes and spun at 1500 × *g* for 15 min at room temperature (RT). Plasma was collected in K2EDTA pre-coated tubes and spun at 2000 × *g* for 15 min at RT. PBMCs were collected in K2-EDTA tubes: blood was diluted in RPMI-1640 without glutamine (Euroclone), layered on Ficoll-Histopaque (Ficoll-PlaqueTM Plus, GE HealthCare) and centrifuged at 800 × *g* for 30 min brake off at RT. PBMCs pellet were collected and washed with PBS without calcium and magnesium (Euroclone). Platelets were eliminated by wash and centrifugation at 200 × *g* for 10 min at RT. Blood samples were stored at − 80 °C in the CCPP Biobank.

### Albumin and IgG removal from serum

Albumin and IgG were removed from serum by using the ThermoScientific^TM^Pierce^TM^ Removal Kit^37^. ≈ 500 µg of serum diluted in “Binding/Wash Buffer” were loaded onto a spin column previously immobilized by the “Cibacron Blue/Protein A gel” and incubated 10 min at RT on an orbital shaker. The samples were centrifuged 1 min at 10,000 × *g* at RT and the recovered filtrate was re-applied on the resin bed. After 10 min of incubation suited by 1 min of centrifugation at 10,000 × *g* at RT, 75 µL of “Binding/Wash Buffer” were loaded to the column and samples were recovered after 1 min of centrifugation at 10,000 × *g* at RT. The column was washed with 100 µL of “Binding/Wash Buffer” and the flow through recovered in 100 µL of Laemmli Loading Buffer 1×.

### Western blotting

Determination of the protein concentration was performed by Coomassie Protein Assay (Thermo Scientific). All samples were diluted in Laemmli Loading Buffer (WVR Life Science). The immunoreactivity signals were detected by Super Signal^TM^West Femto Maximum Sensitivity Substrate (Thermo Scientific) using: two different rabbit polyclonal anti-STX-1a, rabbit polyclonal anti-ERp57, rabbit polyclonal anti-CD9, rabbit polyclonal anti-SNAP-25; rabbit polyclonal anti-Lamin A/C, rabbit monoclonal anti-SV2A, rabbit monoclonal anti-GFAP, rabbit monoclonal anti-PSD95, mouse monoclonal anti-β-actin (ACTB), rabbit polyclonal anti-APO A1, rabbit polyclonal anti-NSE, mouse monoclonal anti-GM130 and mouse monoclonal anti-CD107a. The informations of all antibodies used have been reported in Supplementary table 1 online. Images were acquired using Azure C300 Gel Imaging System (Bio-System) and WB densitometric analyses were performed using Image J software (Meida Cybernetics).

### Cells cultures

After isolation, cells have been washed in DMEM (Biowest LLC, without FBS, Corning) and resuspended in DMEM with 10% FBS and 1% penicillin/streptomycin. 500 µL of cells suspension (1 × 10^6^ cells/mL) have been seeded in a 24 well cell culture plate. Cells adhering to the plate are the monocyte population whereas the floating one are lymphocytes^38^. At each time-point analyzed, cells were harvested and resuspended in Laemmli Loading Buffer (1 × 10^4^ cells/µL), sonicated and 20 μL of each sample has been used for WB analyses.

The SH-SY5Y human glioblastoma cells (ATCC CRL-2266) were maintained in DMEM with 10% FBS, 2 mM l-glutamine and 1% penicillin/streptomycin at 37 °C in a 5% CO_2_ incubator.

### Immunofluorescence experiments

For immunostaining of PBMCs, round glass coverslips were coated with poly-l-lysine 0.1 mg/mL solution (Serva Electrophoresis GmBH). Frozen PBMCs were resuspended in 100 µL of PBS and seeded for 1 h on coated coverslips at 4 °C. Cells were fixed in 1% p-formaldehyde (Thermo Scientific) 15 min at 4 °C.

PBMC’s colocalization analysis between STX-1a and β-actin or GM130 were performed on 5 HC subjects, while measurement of STX-1a expression levels in 5 IS and 5 HC subjects.

SH-SY5Y cells were seeded on round glass coverslips and incubated for 24 h, then washed and fixed with 4% p-formaldehyde for 15 min at 4 °C.

Both cell types were permeabilized, blocked and incubated O.N. at 4 °C with primary antibodies: two different rabbit polyclonal anti-STX-1a, rabbit polyclonal anti-SNAP-25, mouse monoclonal anti-β-actin and mouse monoclonal anti-GM130. The informations of all antibodies used have been reported in Supplementary table 1 online. Cells were then washed and incubated with anti-rabbit TRITC and anti-mouse FITC (1:400, Jackson Immuno Research) followed by incubation with 0.1 µg/mL of DAPI (PanReac AppliChem).

### Fluorescence microscopy and image analysis

Fluorescent images were acquired using NIS-Elements Basic Research software using a fluorescent microscope (Nikon Eclipse Ti2-E). Image J software was used to evaluate fluorescent intensity, in at least 15 isolated cells for each subject and the mean was calculated by the analysis of 5 subjects for each group.

The colocalization parameters were estimated by measuring the percentage of colocalized area and Pearson’s coefficient. In Pearson’s coefficient analysis the JACoP plugin was used.

For the quantification of STX-1a levels fluorescence intensity of nuclei, cytoplasm and STX-1a were calculated. The quantification of STX-1a levels was obtained by dividing the values of its signal to that of β-actin subtracted to nucleus fluorescence.

### Subcellular fractionation

Cellular pellets were lysed in hypotonic buffer (10 mM Hepes/NaOH pH 7.5, 250 mM sucrose, 10 mM NaCl, 3 mM MgCl2, 0.5% Triton X-100, protease and phosphatase inhibitors, from Thermo Scientific) and cytoplasmic fractions were separated from nuclei by 5 min centrifugation at 2000 × *g* at 4 °C. Pellets were resuspended in 100 mM Tris–HCl pH 7.4, 1 mM EDTA pH 7.5 and 500 mM NaCl, protease and phosphatase inhibitors, incubated 10 min at 4 °C, diluted (1:10) and incubated 15 min at 4 °C with 10 mM Tris–HCl pH 7.4, 1 mM EDTA pH 7.5, 0.5% NP-40, protease and phosphatase inhibitors and centrifuged 5 min at 150 × *g* at 4 °C^39,40^. Both fractions were precipitated by the addition of 9 volumes of ethanol, cooled 2 h at − 80 °C and then centrifuged for 30 min at 18,400 × *g* at 4 °C. Pellets were resuspended in 30 µL of Laemmli Loading Buffer 1× and 4 µL of each sample were loaded for WB analysis.

The analysis of cytosolic STX-1a levels were performed in 10 IS, 10 HC and 5 Y-HC subjects.

### Extracellular vesicles purification

500 µL of serum was added to 500 µL of PBS containing three times the suggested concentrations of protease and phosphatase inhibitors and were spun at 4000 × *g* for 20 min at 4 °C. Supernatants were mixed with 250 µL of ExoQuick (System Biosciences), incubated 1 h at 4 °C and centrifuged at 1500 × *g* for 20 min at 4 °C. The pellets were resuspended in 500 µL of Ultra-Pure Water (Lonza Bioscience Solution) with protease and phosphatase inhibitors and incubated for 2 h at RT. Then, 100 µL of samples were stored (total extracellular vesicles proteins, T). NDEs were immunoprecipitated with 4 μg of mouse anti-human CD171 (L1 cell adhesion molecule [L1CAM]) biotinylated antibody from eBioscience) in 45 μL of 3% BSA in PBS and incubated 1 h at 4 °C in a rotating wheel and then incubated with 13.5 μL of streptavidin-agarose Ultralink resin (Thermo Scientific) in 40 μL of 3% BSA for 30 min at 4 °C on a rotating wheel. Samples were spun at 200 g for 10 min at 4 °C. Supernatants represent the total extracellular vesicles fraction depleted of NDE (T–N), while pellets were resuspended in 160 µl of 0.1 M Glycine, pH 2.5–3, centrifuged at 4500 × *g* for 5 min at 4 °C. Supernatants, which represent NDEs fractions (N), were added of 13.5 μL of 1 M Tris–HCl, pH 8, 22.5 μL of 3% BSA in PBS and 234 μL of RIPA Buffer containing protease and phosphatase inhibitors^41^. Samples were subjected to 2 freeze–thaw cycles and sonication.

The analysis of STX-1a expression levels in NDEs and of SNAP-25 in both total extracellular vesicles and NDE were performed in 10 IS and 10 HC subjects.

### Transmission electron microscopy

Extracellular vesicles were fixed in 2% paraformaldehyde and adsorbed on formvar-carbon-coated copper grids, incubated in 1% glutaraldehyde for 5 min, washed with deionized water, and then stained with 2% uranyl oxalate (pH 7) for 5 min and methyl cellulose/uranyl for 10 min at 4 °C. The grids were observed with a TEM (FEI Morgagni268D) at an accelerating voltage of 80 kV. Digital images were taken with Mega View imaging software^42^.

### Statistical analysis

One-way ANOVA analysis followed by post-hoc Tukey significance tests were used to evaluated the STX-1a and SNAP-25 expression levels (Kaleidagraph software—Synergy Software).

Correlation between clinical scales and protein levels were determined by Pearson’s and Spearman’s correlation coefficient *r* or *r*_*s*_ respectively, with 95% by using Jamovi software (version 1.2). The strength of correlation were classified according to British Medical Journal guidelines^43^*.*

## Results

### STX-1a and SNAP-25 are detected in human peripheral blood

**Figure 1:**
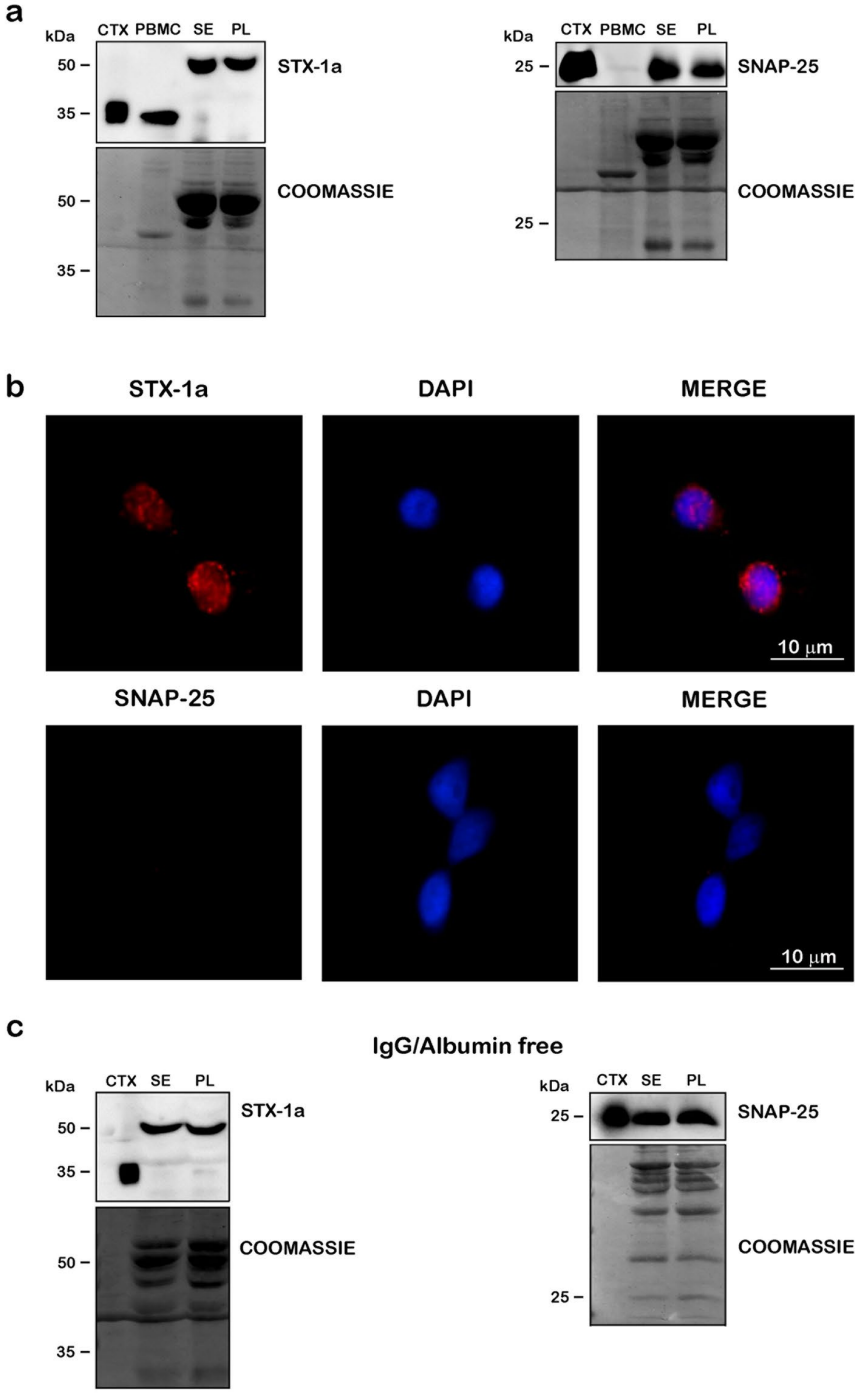
Detection of STX-1a and SNAP-25 in peripheral blood of healthy donors. (**a**) WB analysis of the SNARE proteins in blood components. PBMCs, serum (SE), plasma (PL) and mouse brain cortex lysate (CTX, loaded as positive control) have been immunostained with STX-1a and SNAP-25 antibodies. STX-1a was detected in all blood components, but only in PBMCs at the predicted M.W., while SNAP-25 was observed in serum and plasma but not in blood cells. (**b**) Immunofluorescence analysis of STX-1a and SNAP-25 in PBMCs. Fluorescence analysis of PBMCs confirms the presence of STX-1a but not that of SNAP-25 (red channels). Nuclei have been labeled with the marker DAPI (blue channels). Original magnification: 60×. Bars correspond to 10 µm. (**c**) WB analysis of STX-1a and SNAP-25 in serum and plasma depleted from IgG and albumin. After the stripping of IgG and albumin from serum and plasma (as shown by Coomassie Blue staining) both STX-1a (at the shifted M.W) and SNAP-25 immune-recognition signals persist. For WB analysis 100 µg of blood components, 0.5 µg of mouse brain cortex lysate and 10 µL of flow through have been loaded in each lane. Uncropped WB and coomassie staining in (**a**) and (**c**) have been reported in Fig. S4 online.

In order to confirm the presence of STX-1a and SNAP-25 in human blood fractions, we firstly analyzed, by WB, the different human blood components (i.e. PBMC, serum and plasma) from healthy donors. In PBMC, the band corresponding to STX-1a was found at the predicted molecular weight of 33 kDa and it was comparable to the one observed in the mouse brain cortex lysate loaded as positive control. On the other hand, as expected, no specific signal was detected for SNAP-25 (Fig. 1a). These results have been also confirmed by immunofluorescence analysis in which STX-1a was found expressed around the nuclei (Fig. 1b) similarly to the signal observed in the neuronal-like cells SH-SY5Y, used as positive control (Supplementary Fig. S1a online). On the other hand, SNAP-25 specific signal was completely absent in PBMC (Fig. 1b), thus supporting our WB results. We also confirmed STX-1a presence in PBMCs by using an antibody specific for the isoform 1a in immunofluorescence experiments (Supplementary Fig. S1b online).

Both STX-1a and SNAP-25 proteins were expressed in serum and plasma, although the immunoreactive band for STX-1a appears to be shifted at higher molecular weight (around 50 kDa) (Fig. 1a). The unexpected migration of STX-1a in serum/plasma samples has been confirmed by using another antibody which target a different epitope of the protein (Supplementary Fig. S1c online). Still, further investigations are needed to elucidate the higher STX-1a band entity.

The analysis of human blood samples is often complicated by the presence of high concentrations of albumin and IgG, which can count for 70% of total serum proteins^37^. For this reason, serum and plasma samples have been re-probed after the IgG and albumin removal. Indeed, both STX-1a (at the higher molecular weight) and SNAP-25 signals were still observed (Fig. 1c). Flow through samples coming from the IgG/albumin removal were analyzed and they showed very faint bands for both proteins (Supplementary Fig. S1d online) confirming the antibodies specificity for the proteins probed.

### Subcellular localization of STX-1a in PBMCs from healthy donors

**Figure 2:**
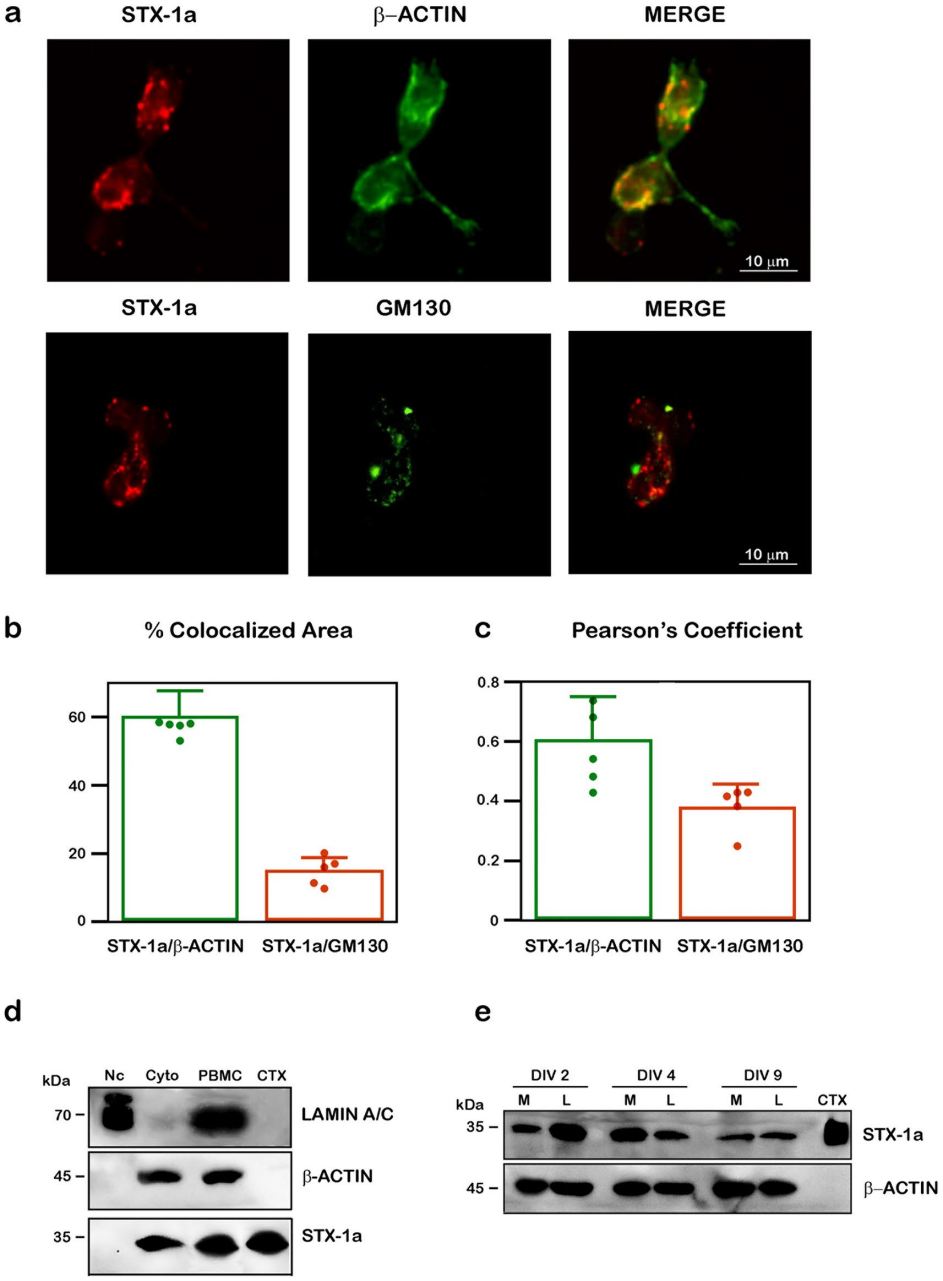
Analysis of the subcellular and cellular localization of STX-1a in PBMCs of healthy donors. (**a**) IF analysis of the cellular distribution of STX-1a in PBMCs. PBMCs have been immunostained with the cytoplasmic marker β-actin (upper panel) or the Golgi apparatus marker GM130 (lower panel) (green channels) and STX-1a (red channels). In the merge images, a clear colocalization of STX-1a with β-actin is evident thus indicating a cytoplasmic localization of the SNARE protein in PBMCs. A partial colocalization of STX-1a and GM130 is also shown. Original magnification: 60×. Bars correspond to 10 µm. (**b**) Colocalization analysis. Histogram showing the percentage of colocalized area compared to the total fluorescent area. (**c**) Pearson’s Coefficient. Histogram showing Pearson’s Coefficient. Green points and histograms represents the % of colocalization area (**b**) and the Pearson’s Coefficient (**c**) of STX-1a compared to β-actin, orange points and histograms represents the % of colocalization area (**b**) and the Pearson’s Coefficient (**c**) of STX-1a compared to GM130. (**d**) PBMC’s subcellular fractions analysis. WB analysis confirmed the cytoplasmic localization of STX-1a in PBMCs. The successful separation of the nuclear (Nc) and the cytoplasmic fractions (Cyto) of PBMCs is demonstrated by the presence of the nuclear marker lamin A/C only in the nuclear fraction and that of β-actin only in the cytoplasmic one. (**e**) STX-1a is expressed in both major PBMCs subtypes in cultures. WB analysis reveals that STX-1a is expressed in both monocytes (M) and lymphocytes (L) and that its expression is maintained over time in cultured cells. For IF analysis at least 15 cells for each subject (n = 5) have been analyzed. For WB analysis 4 µL of PBMCs subcellular fractions (i.e. Nc and Cyto), 100 µg of whole PBMCs, 0.5 µg of mouse brain cortex lysate and 2 × 10^5^ of cultured PBMCs have been loaded in each lane. Bars represent the median value ± SD. Uncropped WB in (**d**) and (**e**) have been reported in Fig. S5 online.

Fluorescence images demonstrated that STX-1a is mostly expressed outside the nucleus (Fig. 1b) with a higher localization in the cytoplasm where strongly colocalizes with the cytoplasmic marker β-actin (Fig. 2a). This interaction is also confirmed by the colocalization areas (60.0 ± 7.7%; Fig. 2b) and Pearson’s Coefficient (0.605 ± 0.141; Fig. 2c) analyses. Moreover, we observed a partial colocalization of STX-1a and the specific marker of the Golgi’s apparatus (GM130) (Fig. 2a) of which the colocalization area (14.8 ± 4.2%; Fig. 2b) and the Pearson’s Coefficient have been calculated (0.380 ± 0.076; Fig. 2c). These results suggest that STX-1a could be synthesized by PBMCs rather than been uptaken from outside.

Then, to confirm the cytosolic localization of STX-1a in PBMCs we performed a subcellular fractionation experiment in which we found that STX-1a was present in the soluble cytoplasmic fraction but not in the nuclear compartment. β-Actin and lamin A/C have been used respectively as markers of cytoplasmic and nuclear compartments to test the quality of the fractionation method (Fig. 2d).

PBMCs chiefly consists of monocytes and lymphocytes, therefore, we finally analyzed whether STX-1a was expressed in both cellular subtypes or one specific sub-population. For this reason, PBMCs have been cultured for 9 days, the longest time point possible before they died, and the expression of STX-1a was monitored at different time points showing that it was expressed in both monocytes and lymphocytes at each time analyzed (Fig. 2e).

### STX-1a and SNAP-25 are detected in NDEs purified from human serum of healthy donors

**Figure 3:**
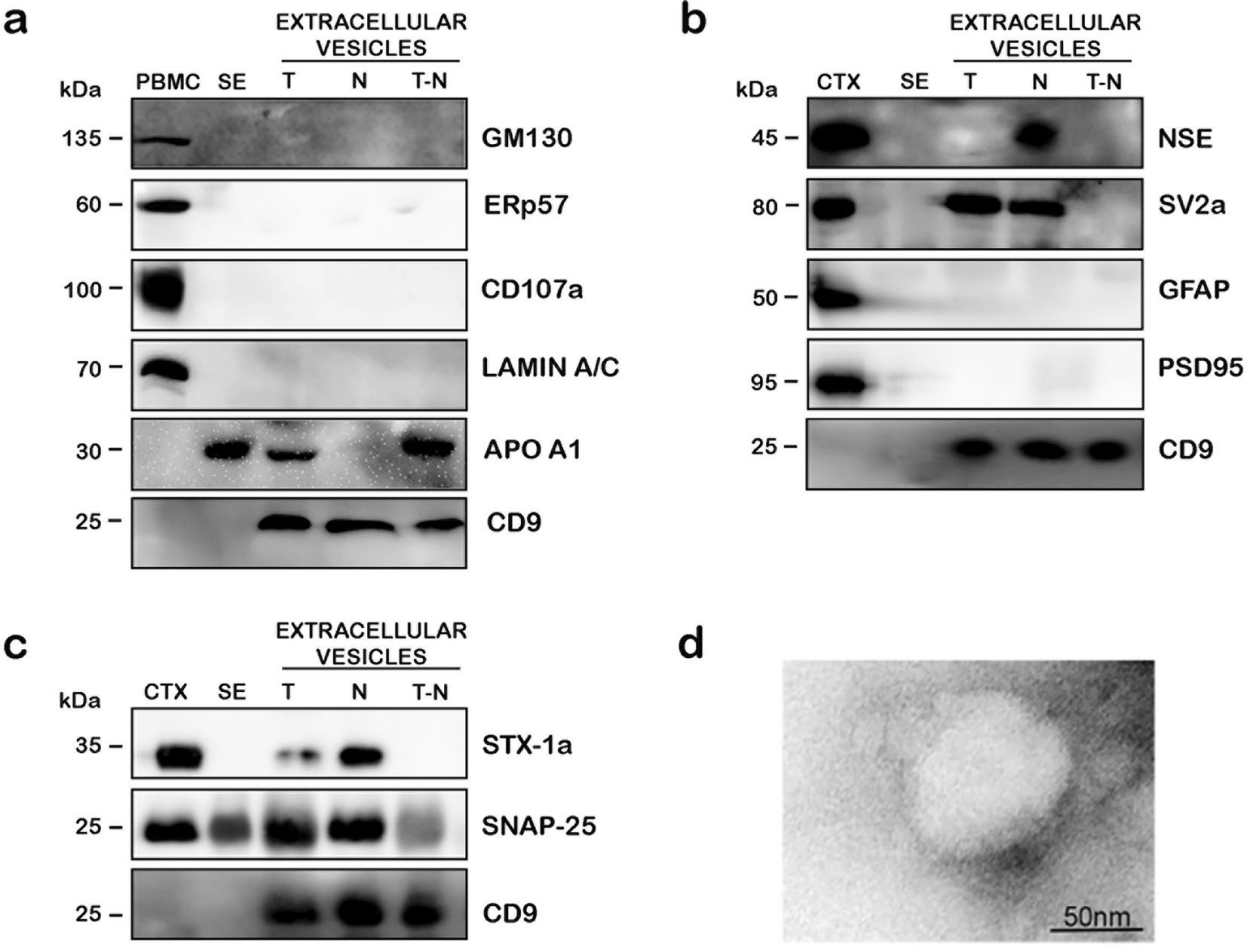
WB analysis of a representative NDE purification from the serum of healthy donors. (**a**) Quality analysis of extracellular vesicle preparation. Extracellular vesicles samples are not contaminated by Golgi apparatus (GM130), endoplasmic reticulum (Erp57), lysosomes (CD107) and nucleus (lamin A/C). The presence of the HDL marker APO A1 is detectable in both total extracellular vesicles (T) and total extracellular vesicles depleted to NDE (T-N), but disappear in NDEs fractions (N). CD9 is a common exosome marker. (**b**) NDE characterization. WB analysis show an enrichment of neuronal markers (NSE and SV-2a) in NDEs with respect to the other two extracellular vesicles fractions. Moreover, NDE samples are negative to the astrocytes and the post synaptic markers GFAP and PSD95, respectively. (**c)** STX-1a and SNAP-25 are enriched in NDE fractions. The levels of both SNARE proteins are increased in NDE with respect to the two other extracellular vesicles fractions. (**d**) Representative EM analysis of NDE. The ultrastructural analysis of NDE shows the presence of rounded-shaped vesicles with a diameter range of 70–100 nm. Bars correspond to 50 nm. For WB analysis 100 µg of whole PBMCs, 50 µg of serum (SE), extracellular vesicles fractions (T, N, T-N) and 0.5 µg of mouse brain cortex lysate have been loaded in each lane. Uncropped WB in (**a**)–(**c**) have been reported in Fig. S6 online.

NDEs were purified from the serum of healthy donors in order to confirm the presence of the two SNARE proteins. The fraction prepared are total extracellular vesicles (T), the NDEs (N) and the total fraction depleted of the NDEs (T-N). Firstly, we examined our preparations in order to verify their quality, indeed all extracellular vesicles fractions resulted pure and free from contaminations by other cells organelles such as Golgi apparatus (GM130), endoplasmic reticulum (ERp57), lysosomes (CD107a) and nucleus (lamin A/C) (Fig. 3a). Moreover, extracellular vesicles preparation resulted positive to CD9 antibody, specific for exosomal population, while it was not present in PBMCs, serum and mouse brain cortex (Fig. 3a–c). Lipoprotein particles are very abundant in the circulation and are often co-purified with extracellular vesicles fractions^44^. In our preparations, the WB analysis showed that the subsequential purification steps succeeded to obtain NDE fractions (N) with a reduced contamination of apolipoproteins, if compared to the other two extracellular vesicles fractions (T and T-N) (Fig. 3a). The purified NDEs fractions, which are enriched of neuronal proteins, were positively immunoreactive to main neuronal markers NSE and SV-2a (Synaptic Vesicle Glycoprotein 2a), but negative to the astrocyte’s marker GFAP and to the post-synaptic marker PSD95 (Post Synaptic Density Protein 95) (Fig. 3b), thus confirming the neuronal origin of these vesicles, potentially from the presynapses. Then, we probed the NDEs for both STX-1a and SNAP-25. Interestingly, STX-1a was present, at the typical molecular weight of 33 kDa, in T extracellular vesicles and also in NDE but not in T-N. In particular, STX-1a appears significantly enriched in NDEs fractions with respect to that of T extracellular vesicles, while it was undetectable in the fractions of T-N. SNAP-25 was present in T and N extracellular vesicles fractions and in less extent also in T-N (Fig. 3c).

Finally, the NDE preparation underwent TEM analyses showing the presence of nano-sized, rounded-shaped vesicles with a typical diameter range of 70–100 nm which can definitely correspond to serum isolated NDEs (Fig. 3d).

### Demographic and clinical characteristics of the human subjects selected for the study

A population of 30 IS patients (mean age 70.0 ± 11.5; 50% females) and 30 age-and-sex matched healthy controls (HC, mean age 72.1 ± 7.3; 50% females) has been selected to study the expression levels of STX-1a and SNAP-25 in serum, PBMCs and NDEs isolated from serum. Being the SDS-PAGE (sodium dodecyl sulphate–polyacrylamide gel electrophoresis) migration of the serum STX-1a at a molecular weight higher than normal, we decided to analyze the expression levels of STX-1a only in PBMCs and NDEs, while being SNAP-25 absent in PBMCs, we analyzed it in serum and NDEs. Moreover, 15 healthy young subjects (Y-HC, mean age 32.6 ± 4.0; 60% females) have been enrolled in the study to assess whether the expression levels of STX-1a and SNAP-25 varied during aging. Table 1 presents the demographic and clinical features of the population studied.

**Table 1:** Study population and clinical data.

Population Characteristics			
	IS patients	HC subjects	Young HC subjects
N°	30	30	15
Males/Females	15/15	15/15	6/9
Age (mean ± SD; range)	70.0 ± 11.5 (45–87)	72.1 ± 7.3 (51–87)	32.6 ± 4.0 (26–39)
TES (%)	26.7	n.a.	n.a.
ACS (%)	46.7	n.a.	n.a.
PCS (%)	26.7	n.a.	n.a.
MMSE (mean ± SD; range)	25.6 ± 8.8 (0–30)	29.1 ± 1.4 (25–30)	n.c.
NIH-SS (mean ± SD; range)	4.9 ± 2.8 (2–14)	n.a.	n.a.
BI (mean ± SD; range)	59.4 ± 20.0 (25–95)	n.a.	n.a.
CIRS (mean ± SD; range)	2.94 ± 1.7/1.54 ± 0.32 (1–9/1.5–2.8)	0.47 ± 0.68/0.98 ± 0.53 (0–2/0–1.7)	0/1.03 ± 0.05 (0/1–1.1)
Processing days after IS (mean ± SD; range)	30.3 ± 12.1 (14–51)	n.a.	n.a.

TES, Thrombo-Embolic Stroke; ACS, Anterior Circulation Stroke; PCS, Posterior Circulation Stroke; MMSE,, Mini-Mental State Examination; NIH-SS, National Institutes of Health Stroke Scales; BI, Bartel Index; n.c., not calculated.

Age or gender distribution was homogeneous between the IS and the HC population groups. Patients suffering from transient ischemic attack (TIA) and hemorrhagic stroke have been excluded from the study. Blood samples of all IS patients were obtained between 14 and 51 days (mean 30.3 ± 12.1 days) from the ischemic event, which is considered the beginning of the post-acute phase of IS, and the majority of the stroke events had origin in the anterior circulation (46.7%). Anova analyses demonstrate no correlation between the time of blood collection in IS patients and NIH-SS (p = 0.786, Supplementary Fig. S2online). The average of the specific stroke clinical scales, i.e. NIH-SS and the modified BI were 4.9 ± 2.8 and 59.4 ± 20.0 respectively, while MMSE is 25.6 ± 8.8 vs 29.1 ± 1.4 of the HC population.

### Peripheral levels of STX-1a and SNAP-25 were significantly increased in IS patients compared to HC subjects and STX-1a correlates with IS clinical scales

**Figure 4:**
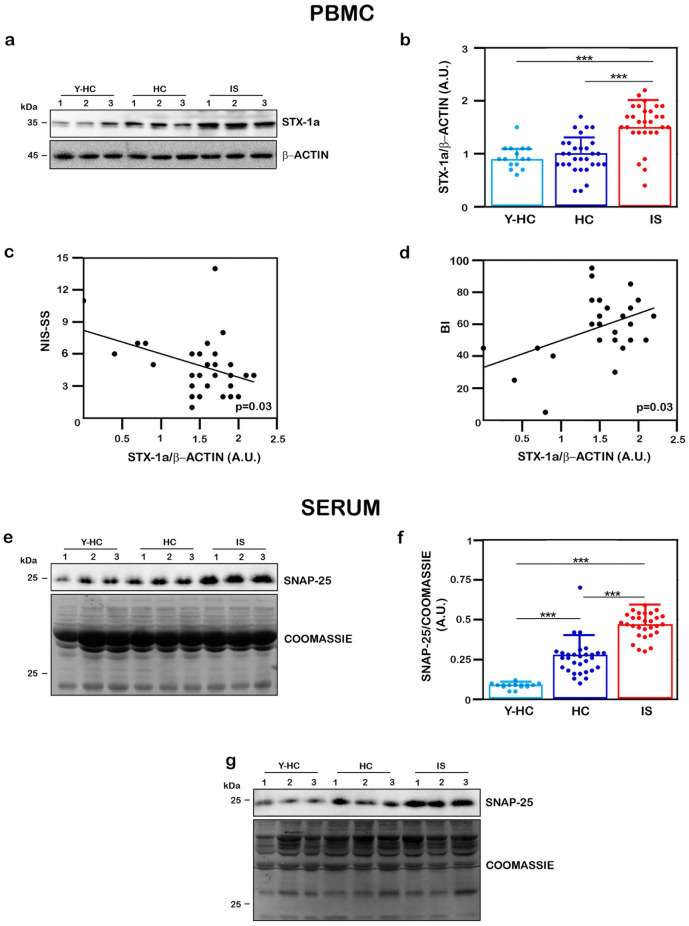
Quantitative analysis of STX-1a in PBMCs and of SNAP-25 in sera in all population analyzed. (**a**, **e**) Representative WB analysis of STX-1a in PBMCs (**a**) and of SNAP-25 in serum (**e**) in IS and HC subjects. PBMCs are immunostained for STX-1a (**a**) and serum for SNAP-25 (**e**). For each sample, the STX-1a densitometric value is normalized to that of β-actin, while the SNAP-25 densitometric values is normalized to that of total blood proteins content revealed by Coomassie Blue staining. (**b**) Densitometric analysis STX-1a expression levels in PBMCs. STX-1a expression levels have been analyzed by using One-way ANOVA with post-hoc Tukey test: STX-1a significantly increase in PBMCs of IS patients with respect to HC and Y-HC subjects (p ≤ 0.0001), while no differences are observed between control groups (p = 0.97). (**c**, **d**) Pearson’s correlation analysis between expression of STX-1a in IS PBMC and the clinical stroke scales. STX-1a levels in PBMCs of IS patients present a moderate negative correlation with NIH-SS (p = 0.03) (**c**) and a moderate positive correlation with BI (**d**) (p = 0.03). (**f**) Densitometric analysis SNAP-25 expression levels in sera. SNAP-25 expression levels have been analyzed by using One-way ANOVA with post-hoc Tukey test: SNAP-25 levels are significantly increased in serum of IS patients with respect to HC and Y-HC subjects (p ≤ 0.0001). A significant increase of SNAP-25 expression is also observed in HC with respect to Y-HC subjects (p ≤ 0.0001). (**g**) WB analysis of SNAP-25 in sera depleted of IgG and albumin. The increased SNAP-25 levels in IS patient are not influenced by the presence of serum proteins. For WB analysis 100 µg of whole PBMCs and of serum (SE) have been loaded in each lane. In quantitative graphs (panels **b** and **f**) each point in a frame depicts the value for a single subject (n = 30 IS patients, 30 HC subjects and 15 young HC subjects) while bars represent the median value ± SD. ***p ≤ 0.001. In panels (**b**) and (**f**) light blue points and histograms correspond to Y-HC subjects, blue points and histograms correspond to HC subjects, red points and histograms correspond to IS patients. Uncropped WB and coomassie staining in (**a**), (**e**) and (**g**) have been reported in Fig. S7 online.

First of all, the expression levels of STX-1a in PBMCs and of SNAP-25 in serum have been analyzed by WB analysis and subsequent densitometric measurement in IS patients, HC and Y-HC subjects. Our analyses revealed that both SNARE proteins significantly increased in IS patients compared to either HC or Y-HC (Fig. 4). We also confirm the “altered” migration of STX-1a in sera of IS patients (around 50 kDa, Supplementary Fig. S3a online) and the concomitant absence of SNAP-25 in their PBMCs (Supplementary Fig. S3b online).

Interestingly, the densitometric analyses showed an increased expression level of STX-1a in PBMCs of IS patients compared to both HC and Y-HC subjects (1.5 ± 0.5 vs 0.97 ± 0.3 and 0.94 ± 0.2 for HC and Y-HC, respectively, p = 0.0002; Fig. 4a, b). We then analyzed a possible correlation between protein expression level in PBMCs and the NIH-SS, BI and MMSE clinical scales. By using Pearson’s correlation analyses, we found a moderate negative correlation with NIH-SS scale (-0.407) (Fig. 4c) and a moderate positive correlation with BI score (0.400) (Fig. 4d). Both these correlations are statistically significant (p = 0.03). These associations were also calculated with the Spearman correlation analyses which confirmed the correlation, but resulting weak (− 0.314, p = 0.05 for NIH-SS and 0.362, p = 0.03 for BI) (Supplementary Fig. 3c, d online). On the other hand, no significant correlation has been found between STX-1a concentration measured in IS PBMCs and MMSE (Supplementary Fig. S3e).

Moreover, the expression levels of SNAP-25 have been found significantly increased in serum of IS subjects with respect to both HC and Y-HC subjects (0.47 ± 0.07 vs 0.28 ± 0.12 and 0.09 ± 0.02 for HC and Y-HC, respectively, p = 0.00004; Fig. 4e, f). Of note, in this case, SNAP-25 is significantly more abundant in the HC population in comparison with the Y-HC subjects. Nevertheless, the Pearson’s analysis between these data and the three aforementioned clinical scales did not lead to any statistically significant results (Supplementary Fig. S3f–h online).

Finally, we demonstrate that the increased SNAP-25 expression levels in IS sera were not affected by the IgG and albumin content since SNAP-25 expression has been observed in serum of the three analyzed population (Fig. 4g), while no signals have been observed in the flow through samples (Supplementary Fig. S3i online).

### STX-1a increased in PBMC samples of a subpopulation of IS patients

**Figure 5:**
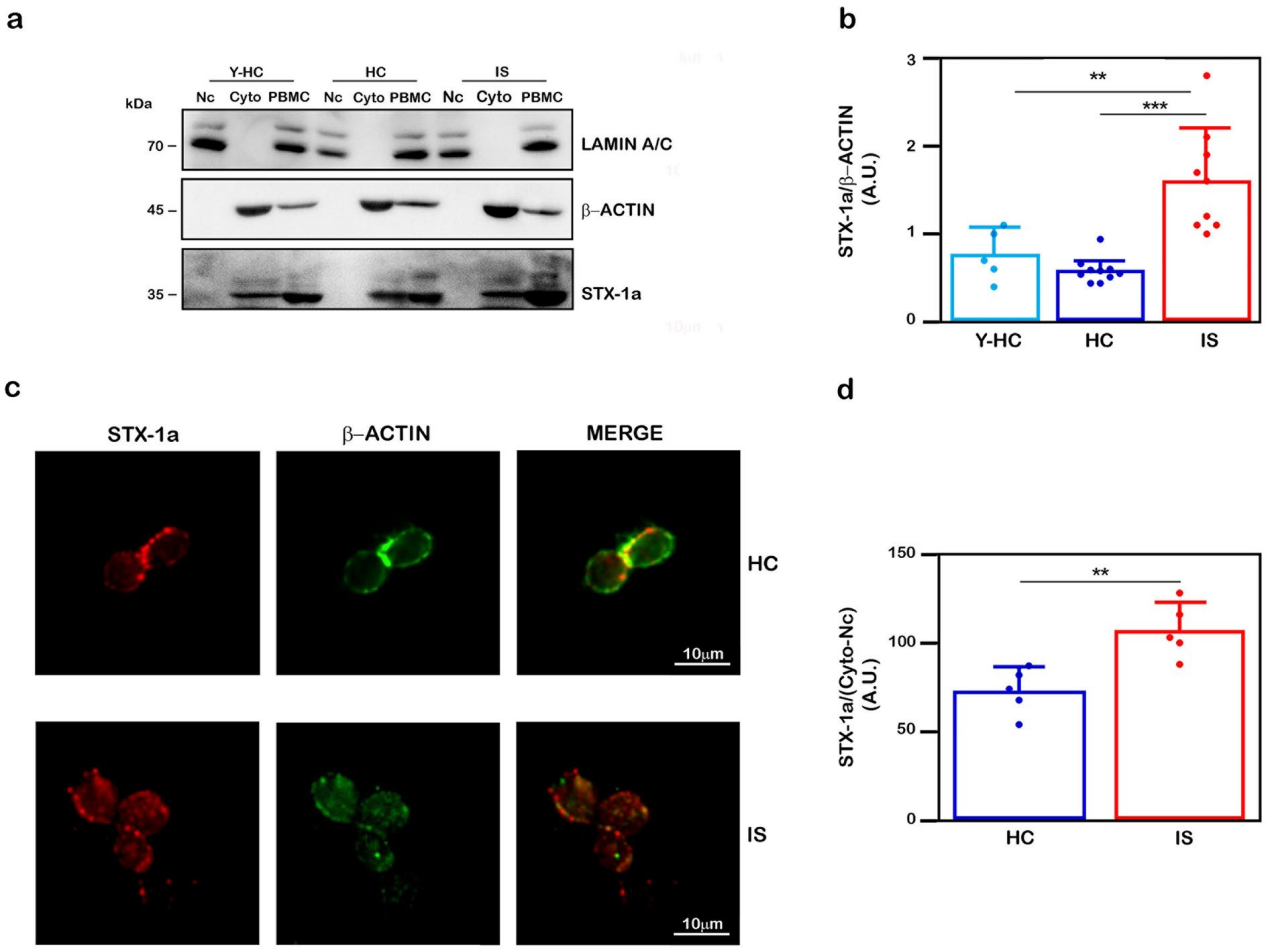
Confirmation of increased STX-1a levels in PBMCs of IS patients with respect to HC subjects. (**a**) Representative WB of STX-1a expression levels in PBMC cytoplasmic fractions. Representative WB analysis of cytoplasmic and nuclear fractions of PBMCs. For each sample, the STX-1a densitometric values have been normalized to that of β-actin. The successful separation of the nuclear (Nc) and the cytoplasmic fractions (Cyto) of PBMCs is demonstrated by the presence of the nuclear marker lamin A/C in the nuclear fraction and that of β-actin in the cytoplasmic one. (**b**) Quantitative analysis of cytoplasmic STX-1a. STX-1a expression levels have been analyzed by using One-way ANOVA with post-hoc Tukey test: STX-1a significantly increase in PBMCs of IS patients with respect to HC (p ≤ 0.0001) and Y-HC (p ≤ 0.003) subjects, while no difference is observed between the two control groups (p = 0.7). (**c**) Representative Immunofluorescence analysis of STX-1a in PBMCs of IS patients and HC subjects. PBMCs have been labeled with β-actin (green) and STX-1a (red). The STX-1a fluorescence intensity has been normalized by dividing the measure of its intensity by that of the β-actin subtracted from that measured for the nucleus (DAPI immunostaining). (**d**) Quantitative analysis STX-1a levels in PBMCs. The normalized STX-1a expression levels have been analyzed by using One-way ANOVA with post-hoc Tukey test: STX-1a levels are significantly increased in PBMCs of IS patients with respect to HC subjects (p = 0.005). Original magnification: 60X. Bars correspond to 10 µm. For WB analysis 4 µL of each fraction (Nc and Cyto) and 100 µg of whole PBMCs have been loaded in each lane. For IF analysis at least 15 cells for each subject have been analyzed. In quantitative graphs (panels **b** and **d**) each point in a frame depicts the value for a single subject (n = 5–10 IS patients, 5–10 HC subjects and 0–5 young HC subjects, respectively in (**b**) and (**d**) while bars represent the median value ± SD. **p ≤ 0.005; ***p ≤ 0.001. Uncropped WB in a) have been reported in Fig. S8 online.

Subcellular fractionation experiments performed on PBMCs from a subpopulation of IS patients, HC and Y-HC subjects confirmed the presence of STX-1a in the cytoplasm of PBMCs in the analyzed cohorts (Fig. 5a). Moreover, STX-1a expression levels have been found significantly higher in the cytoplasm of PBMCs of IS patients with respect to HC subjects (1.6 ± 0.6 vs 0.59 ± 0.1, p = 0.0002; Fig. 5b) and Y-HC (0.77 ± 0.3, p = 0.0027; Fig. 5b). No significant differences were observed between the two control groups, HC and Y-HC (p = 0.70) (Fig. 5b).

The immunofluorescence analysis of PBMCs from a subpopulation of IS patients confirmed a higher presence of STX-1a signal in this group compared to the HC subjects (Fig. 5c). Indeed, the fluorescent analysis of STX-1a expression confirms that STX-1a was significantly increased in IS patients (107 ± 15.4 vs 73.1 ± 12.9, p = 0.005; Fig. 5d).

### STX-1a and SNAP-25 expression levels are significantly increased in NDEs purified from serum of a subpopulation of IS patients

**Figure 6:**
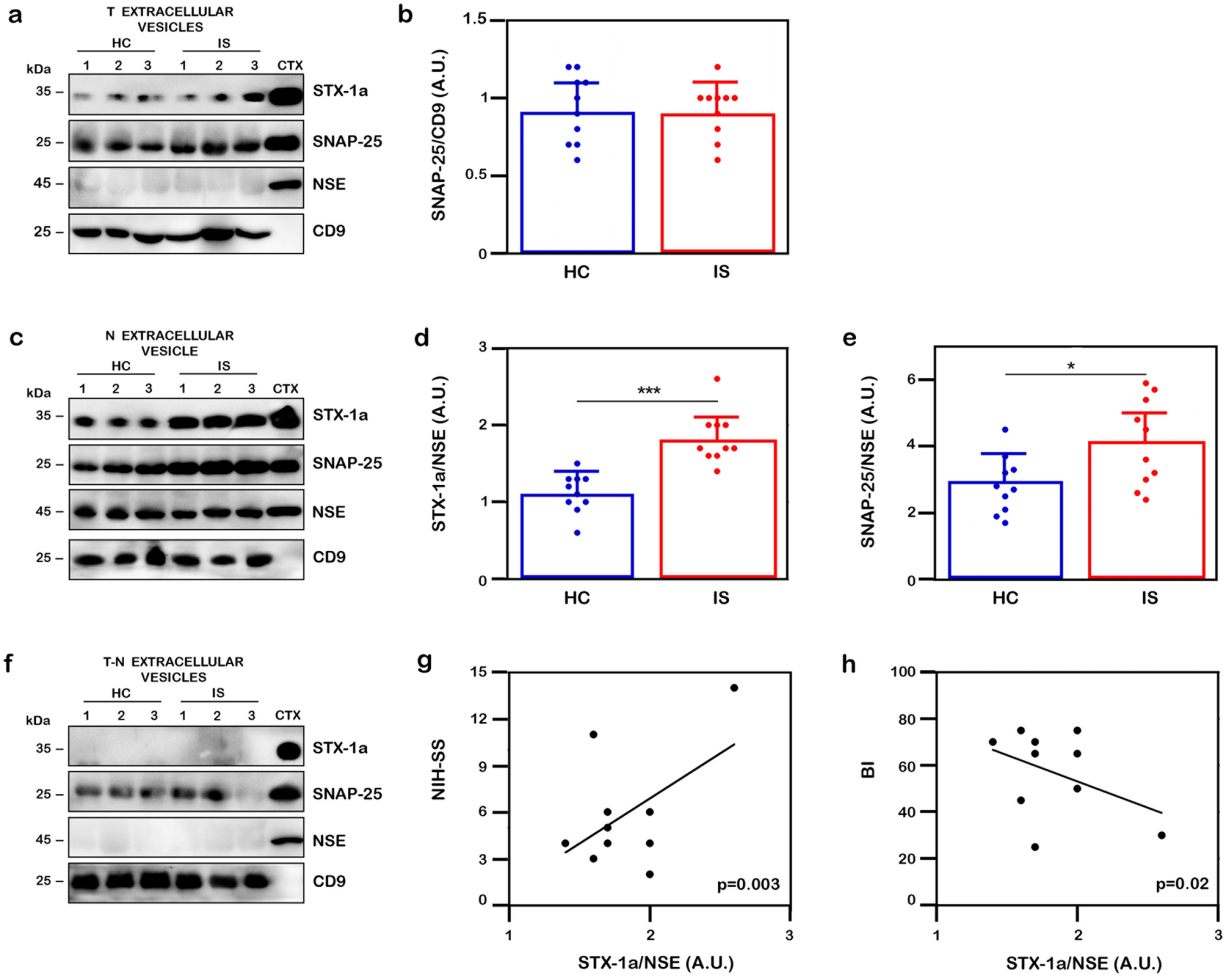
Analysis of SNARE in extracellular vesicles of IS and HC subjects. (**a**, **c**, **f**) Representative WB analysis of the expression levels of STX-1a and of SNAP-25 in extracellular vesicles derived from sera. Representative WB analysis of total (T) extracellular vesicles (**a**), NDE (N) (**c**) and total extracellular vesicles depleted of NDE (T-N) (**f**) fractions. Each sample has been tested against CD9 (total exosomes marker), NSE (NDE marker), STX-1a and SNAP-25. The SNAP-25 densitometric values in total extracellular vesicles fraction have been normalized to that of CD9 (**a**), while the STX-1a and SNAP-25 densitometric values in NDE have been normalized to that of NSE (**c**). (**b**, **d**, **e**) Densitometric analysis of STX-1a and SNAP-25 in extracellular vesicles The normalized SNAP-25 levels in T extracellular vesicles (**b**) and those of both STX-1a (**d**) and SNAP-25 (**e**) in NDE have been analyzed by using One-way ANOVA with post-hoc Tukey test. Both STX-1a and SNAP-25 expression levels are significantly increased in NDE of IS patients with respect to HC subjects (p ≤ 0.0001 and p ≤ 0.02 for STX-1a (**d**) and SNAP-25 (**e**) respectively), while no differences is observed for SNAP-25 in the T fraction (p = 0.91) (**b**). (**g**, **h**) Pearson’s correlation analysis between expression of STX-1a in IS NDEs and the clinical stroke. STX-1a levels in NDE of IS patients present a moderate positive correlation with NIH-SS (p = 0.003) (**g**) and a moderate negative correlation with BI (p = 0.02) (**h**). For WB analysis 50 µg of each extracellular vesicles fraction and 0.5 µg of mouse brain cortex lysate have been loaded in each lane. In quantitative graphs (panels **b**, **d** and **e**) each point in a frame depicts the value for a single subject (n = 10 IS patients and 10 HC subjects) while bars represent the median value ± SD. *p ≤ 0.05; ***p ≤ 0.001. In panels (**b**), (**d**) and (**e**) blue points and histograms correspond to HC subjects, red points and histograms correspond to IS patients. Uncropped WB in (**a**), (**c**) and (**f**) have been reported in Fig. S9 online.

STX-1a and SNAP-25 proteins content have been analyzed in the extracellular vesicles’ fractions isolated from the serum of a subpopulation of IS patients and HC subjects. All extracellular vesicle fractions have been tested for the exosomal marker CD9 and the neuronal marker NSE (Fig. 6). The STX-1a signal was barely visible but not quantifiable in the total extracellular vesicles’ fractions (Fig. 6a), and absolutely absent in the total extracellular vesicles fractions depleted from NDEs (T-N) (Fig. 6f), but it was significantly increased in NDEs derived from IS patients with respect to HC (1.8 ± 0.3 vs 1.1 ± 0.3, p = 0.00005; Fig. 6c, d). On the other hand, SNAP-25 resulted strongly in the total extracellular vesicles fractions compared with STX-1a, but differences of SNAP-25 expression were not significant between the two above-mentioned populations (p = 0.91) (Fig. 6a, b). However, SNAP-25 expression level was statistically higher in NDEs isolated from IS patients than HC subjects (4.1 ± 0.9 vs 2.9 ± 0.9, p = 0.02; Fig. 6c, e).

Finally, SNARE protein contents in NDEs have been correlated with the clinical scales through a Pearson’s correlation analysis and only STX-1a showed significant results. In particular, a moderate positive correlation between STX-1a and NIH-SS scale (0.521) (Fig. 6g) and a moderate negative correlation with BI scores (− 0.431) (Fig. 6h) were found. Both correlations are statistically significant (p = 0.003 and p = 0.02, for NIH-SS and BI scales, respectively). The same analyses performed with the Spearman test resulted in strong correlations (0.65, p = 0.03 for NIH-SS and − 0.622, p = 0.04 for BI) (Supplementary Fig. S4a, b online). On the other hand, no correlation has been observed between STX-1a and MMSE (Supplementary Fig. S4c online) and between SNAP-25 NDE’s concentration and the clinical scales (Supplementary Fig. S4d–f online). Finally, even the levels of the two SNARE proteins in the NDEs are not statistically correlated with each other (Supplementary Fig. S4g online).

## Discussion

The identification of IS blood-based biomarkers would represent a fundamental step for unsure a better prediction of the ischemic event outcomes and for the identification of a personalized treatment for each patient with the advantage to be very little invasive and relative at low-cost. Emerging evidences demonstrate that exosomes regulate brain intercellular communication after IS^45^, for this reason, the development of techniques to isolate NDEs from peripheral blood has opened interesting opportunities in the field of the identification of IS biomarkers^41^. In fact, NDEs have demonstrated to be useful in the diagnosis for neurodegenerative diseases^30,32,41^. Importantly, EVs can cross the blood–brain barrier (BBB) thus, the isolation of EVs enriched for neuronal origin from peripheral blood may dynamically reflect and track neuropathological changes^41^. In addition, PBMC, being present at the peripheral level, can represent another source of IS biomarkers. These cells selectively migrate and infiltrate the ischemic brain tissue^46^ and an alteration of their gene expression profile has been observed in IS patients^47^. Indeed, the destruction of the BBB allows proteins to leak into the blood flow and their serological detection become therefore possible. Interestingly, it has been demonstrated that some cerebral proteins are released and detected into blood samples of IS patients^19^.

In this work, we investigated the IS peripheral expression levels of two proteins member of the SNARE complex, STX-1a and SNAP-25, that play a fundamental role in the release of neurotransmitters from the pre-synaptic side^48,49^ and which concentration is altered in the brain tissue of IS animal models^16–18^. Recently, both proteins have been detected in NDEs isolated from blood serum and the reduction of their expression levels have been associated with Alzheimer and Parkinson diseases^30,32^. Moreover, both proteins have yet been described in blood cells like neutrophils^28,50,51^ and platelets^52,53^, but only the mRNA of STX-1a (but not that of SNAP-25) was detected in PBMC^29^.

Considering the defined critical timepoints post-stroke that link to the currently known biology of recovery^54^, we decided to focus our attention on the early subacute phase (7–60 days), critical time for endogenous plasticity, with the intent to detected synaptic biomarkers reflecting underlying biological mechanisms, according to consensus-based core recommendations from the Stroke Recovery and Rehabilitation Roundtable^55^. However, we are aware that the population analyzed in our work could show some limitation due to the fact that we took in account patients with 30 days (mean) after IS, but to have a more complete frame of the biomarker efficacy in our future studies we will include early and late stage IS population.

First of all, we confirmed the presence of both proteins at peripheral levels and we found that SNAP-25 was present in serum and in both total extracellular vesicles and NDEs lysates, but not in PBMC, as already reported in the literature^29,31^. On the other hand, STX-1a expression was observed in PBMC and NDEs, while in total extracellular vesicles was almost undetectable by WB analyses. These results lead us to speculate that both SNARE proteins might be transported through NDEs in human serum. In addition, the differential pattern of STX-1a and SNAP-25 distribution in EVs (i.e. SNAP-25 in both total and neuronal EVs, while STX-1a only in NDEs) might be due to the higher expression level of SNAP-25 in peripheral blood respect to STX-1a. Although, it was reported that STX1a mRNA is present in PBMC^29^, its protein expression in these cells is here described for the first time. Still, there are no available data about the role of STX1a in PBMC, although it is known that these cells are able to release cytokines^56^, so potentially syntaxins protein family could be useful for the release machinery in these cells. Interestingly, in both serum and plasma samples, and by using two different antibodies recognizing two different epitope of the protein, we found that the molecular band corresponding to STX-1a is shifted to higher molecular weight respect to the classical 33 kDa, this is probably due to post-translational modifications or protein dimerization events for which we will need further investigations.

Regarding the cellular distribution of STX-1a, we observed, by mean of immunofluorescence and subcellular fractionations experiments, that it was expressed mainly in the cytosol. Interestingly, the partial colocalization of STX-1a with the GM130-Golgi apparatus marker led us to assume that STX-1a could be processed by the Golgi organelle during its in-situ syntheses. Moreover, another hypothesis on the cause of the presence of STX-1a in the PBMC could be that it is up-taken by the cells from the quote of the protein freely moving in the blood flow. Interestingly, we demonstrated that both monocytes and lymphocytes, the two main cellular sub-population of PBMC, express STX-1a. In line with our results, syntaxin proteins, but not STX-1a, were reported to play an important role in the cellular release of cholesterol and choline-phospholipids to apolipoprotein A-I (apoA-I) in monocytes by binding ATP-binding cassette transporter A1 (ABCA1)^57^, and in addition, STX-11 was found to regulate lymphocyte-mediated secretion and cytotoxicity^58^.

We analyzed the peripheral expression of STX-1a and SNAP-25 in a cohort of IS patients that has been characterized by the classical IS clinical scales NIH-SS and BI as well as by MMSE^36^ and CIRS^35^ in order to also evaluate their cognitive function as well as to select patients with low comorbidity levels. Our data indicated that in all blood components analyzed, the levels of both SNARE proteins were significantly higher in IS patients compared to the two control populations (HC and Y-HC). In particular, we demonstrated that the level of STX-1a was increased in PBMCs of IS patients and no significant difference was observed between HC and Y-HC subjects leading us to speculate that the expression protein level fluctuation is related to the ischemic event rather than to the aging process. These results are in line with reported data showing that other brain pathologies stimulate up-regulation of proteins in PBMCs as for example happens for α-synuclein in Parkinson’s disease patients^59^ or the increase of mRNAs like IL-1 (Interleukin) beta, IL-8, and IL-17 mRNA in PBMC during brain ischemia^60^. Likewise, SNAP-25 levels were significantly increased in sera of IS patients compared to the age-matched healthy subjects and, interestingly, its expression level seems to be also influenced by aging processes, since HC subjects were characterized by significant higher SNAP-25 serum levels with respect to the younger population. The role of SNAP-25 in aging is so far not explored, however our findings suggest that its peripheral levels could reflect some physiological modification of brain synapses which occurs during the aging.

In addition, both STX-1a and SNAP-25 levels were significantly enriched in NDEs purified from sera of IS patients. The release of neuronal proteins in extracellular vesicles has been reported to show a potential use of their quantification as a biomarker for different pathologies like Alzheimer’s in the case of SNAP-25^30^ and Parkinson’s, for the presynaptic SNARE protein complex (i.e. STX-1a, SNAP-25 and VAMP-2)^32^. Moreover it has been demonstrated that STX-1a plays an important role in the regulation of the release of exosomes from the central nervous system^61^ and, interestingly, other syntaxin isoforms were found to have a role mainly in exosomal secretion from different cell types^62^.

Pearson’s and Spearman’s correlation analysis of STX-1a levels, in both PBMCs and NDEs, lead us to hypothesize that the presence of STX-1a is correlated with the IS pathology since it is associated with NIH-SS clinical scale, which is among the most frequent clinical and demographical scale associate with stroke mortality^33^ and with BI, that measures the performance in activities of daily living^34^. However, we did not observe any correlation with the MMSE, which is not a specific clinical scale for the IS, but an assessment of cognitive functions^36^. Worthy of note, STX-1a levels always correlates with clinical scales, but in an opposite manner in PBMC and NDE. In NDEs, STX-1a levels positively correlate with NHI-SS and negatively with BI indicating that STX-1a expression increases together with the worsen clinical frame of the patients while is the contrary in PBMC. Indeed, our results suggest that STX-1a might be synthesized by PBMC^29^ (the protein partially colocalized with Golgi apparatus and STX-1a mRNA was previously reported in these cells), however its physiological or pathological role has not been elucidated yet. All together, these results demonstrate that STX-1a not only is linked to the pathology onset but also correlated with its severity, being higher in patients with a more severe score in clinical scales. On the other hand, the levels of SNAP-25 never showed any statistical difference when compared with the clinical scales, probably the presence of these proteins is not strictly related to the severity of the IS (indeed, we also observed its blood accumulation in aging), but its peripheral levels increase is anyway associated with brain ischemia pathology.

All these findings prompted us to propose that the observed biological modifications of STX-1a and SNAP-25 could potentially represent the extent of brain damage and, consequently, they could be studied as potential prognostic biomarkers for IS.

Although we used NIHSS, which has been validated as a tool to assess the severity of stroke and as an excellent predictor for patient outcomes^33,63^, a possible limitation of the work carried out is the lack of neuroimaging data related to the population under study. The other limitation of this work is represented by the time of blood sampling after stroke. Therefore, information regarding brain images as well as the analysis of the predictive value of the neurological evolution measured by our biomarkers at earlier and later stage of the pathology will certainly need to be included in future studies.

## Conclusion

Our study demonstrates the SNARE proteins STX-1a and SNAP-25 are present in human blood fractions and to the best of our knowledge, this is the first time that the neuronal STX-1a protein is observed in PBMCs. The analysis of their expression levels showed that both the two SNARE proteins analyzed are augmented in the blood fractions of a cohort of IS patients, HC and Y-HC subjects. Interestingly, the STX-1a increase, in both PBMCs and NDEs, always correlates with the severity of the pathology assessed by using the IS clinical scales NIH-SS and BI. Indeed, these findings might set the basis to study the synaptic plasticity changes, following an ischemic attack, at peripheral level and STX-1a and SNAP-25, together with other markers that will be discovered, measured at early time after IS or in a longitudinal observation, could be considered as potential prognostic biomarkers for understanding the effects of rehabilitation interventions on the population affected by IS.

## Supplementary Information

Supplementary Information 1.

Supplementary Information 2.

## Data Availability

The datasets generated during and/or analyzed during the current study are available from the corresponding author on reasonable request. All data generated or analyzed during this study are included in this published article (and its supplementary information files).

## Abbreviations

ABCA1: ATP-Binding Cassette transporter A1
ApoA-I: Apolipoprotein A-I
ATP: Adenosine Tri-Phospahte
BBB: Blood Brain Barrier
BI: Bartel Index
BSA: Bovine Serum Albumin
CCPP: Casa di Cura Privata del Policlinico
CIRS: Cumulative Illness Rating Scale
DMEM: Dulbecco’s Modified Eagle medium
FBS: Fetal Bovine Serum
GFAP: Glial Fibrillary Acid Protein
HC: Healthy Controls
HSP70: Heat Shock Protein 70
IgG: Immunoglobulin G
IL: Interlerukin
IS: Ischemic Stroke
K2EDTA: Ethylenediaminetetraaceticacid-K2
L1CAM: L1 Cell Adhesion Molecule
Lp-PLA 2: Lipoprotein-associated Phospolipase A2
MMP-9: Matrix Metalloproteinase 9
mRNA: Messenger Ribonucleic Acid
MMSE: Mini-Mental State Examination
NDE: Neuronal Derived Extracellular Vesicles
NIH-SS: National Institutes of Health Stroke Scale
NSE: Neuron Specific Enolase
O.N.: Over Night
PBMC: Peripheral Blood Mononuclear Cells
PBS: Phosphate Buffered Saline
PSD95: Post Synaptic Density Protein 95
PVDF: Polyvinylidene difluoride
RIPA: Radioimmunoprecipitation Assay Buffer
RPMI: Roswell Park Memorial Institute culture medium
R.T.: Room Temperature
S100β: S100 calcium-binding protein B
SDS-PAGE: Sodium dodecyl sulphate–polyacrylamide gel electrophoresis
SNAP-25: Synaptosomal Associated Protein, 25 kDa
SNARE: Soluble N-Ethylmaleimide-Sensitive Fusion Protein Attachment Protein Receptor
STX: Syntaxin
SV-2a: Synaptic vesicle Glycoprotein 2a
TIA: Transient Ischemic Attack
TBS: Tris-Buffered Saline
TEM: Transmission Electron Microscopy
WB: Western Blot
Y-HC: Young Healthy Controls
