# Supplementary material for: Syntaxin-1a and SNAP-25 expression level is increased in the blood samples of ischemic stroke patients
Source: Sci Rep. 2022 Aug 25;12:14483. doi: 10.1038/s41598-022-18719-2 (PMC9411545; doi:10.1038/s41598-022-18719-2)
Supplement: Supplementary file 2 — Supplementary Information 2. [file 41598_2022_18719_MOESM2_ESM.doc]

**Supplementary file**

**Syntaxin-1a and SNAP-25 expression level is increased in the blood samples of ischemic stroke patients.**

Pamela Cappelletti^1^, Melania Filareti^1^, Laura Masuelli^2^, Roberto Bei^3^, Kambiz Hassanzadeh^4,#^, Massimo Corbo^1^, Marco Feligioni^1,4*^

^1^ Department of Neuro-rehabilitation Sciences, Casa Cura Policlinico, Milan, Italy;

^2^Department of Experimental Medicine, University of Rome “Sapienza”, Rome, Italy

^3^Department of Clinical Sciences and Translational Medicine, University of Rome “Tor Vergata”, Rome; Italy

^4^EBRI Rita Levi-Montalcini Foundation, Rome

**^#^**current address: Fondazione Pisana per la Scienza (FPS), Pisa

*Corresponding author: Marco Feligioni, PhD

**Correspondence**:

Marco Feligioni, PhD

European Brain Research Institute (EBRI) Rita Levi Montalcini Foundation,

Viale Regina Elena 295, 00161 Rome, Italy

Phone: +39 06 49 255 255

Fax: +39 06 49 255 255

e-mail: [m.feligioni@ebri.it](mailto:m.feligioni@ebri.it)

**
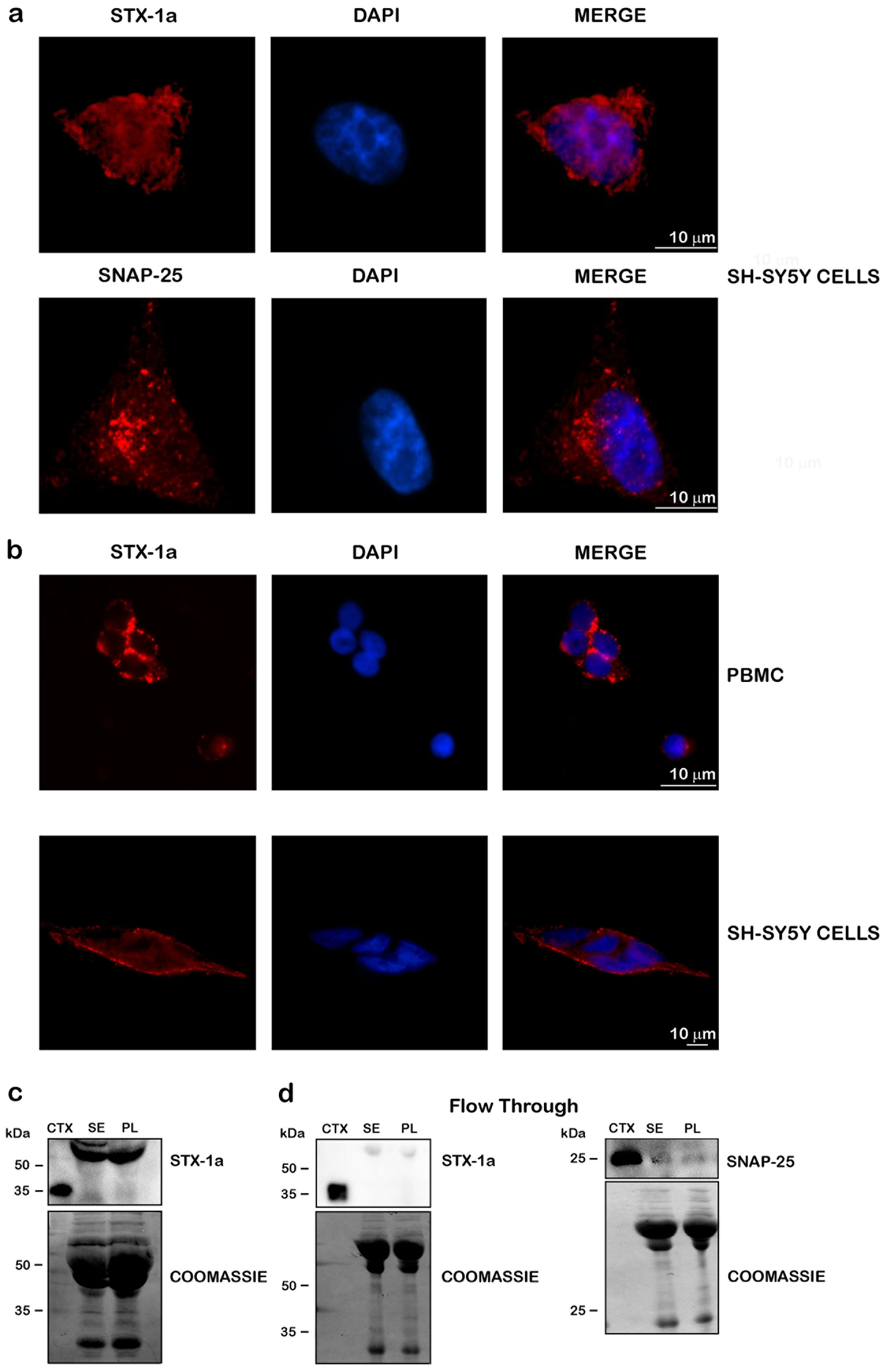
**

**Fig. S1 Analysis of STX-1a and SNAP-25 in SH-SYS5 cell line and in peripheral blood of healthy donors.** **a) IF analysis of STX-1a and SNAP-25 in SH-SY5Y cells line.** Immunostaining of SH-SY5Y cells, for both synaptic proteins (red channels), has been performed as positive control. **b) STX-1a presence in PBMCs by IF analysis with an additional antibody.** Immunostaining of PBMC for STX-1a (red channel) through an antibody specific for the isoform 1a. DAPI (blue) has been used to stain the cells nuclei. 60X magnification with bars corresponds to 10 µm. **c) STX-1a altered migration in WB analysis confirmed with an additional antibody.** Stx-1a was detected at the shifted molecular weight by using of another specific antibody. **d) WB analysis of STX-1a and SNAP-25 in the flow through of serum and plasma depleted from IgG and albumin.** After the stripping of IgG and albumin from serum and plasma no specific signal is detected in the flow through for both STX-1a and SNAP-25.

For WB analysis 0.5 µg of mouse brain cortex lysate, 100 µg of serum and plasma, and 10 µL of flow through has been loaded in each lane. Uncropped WB and coomassie staining in c and d) have been reported in Fig. S11.


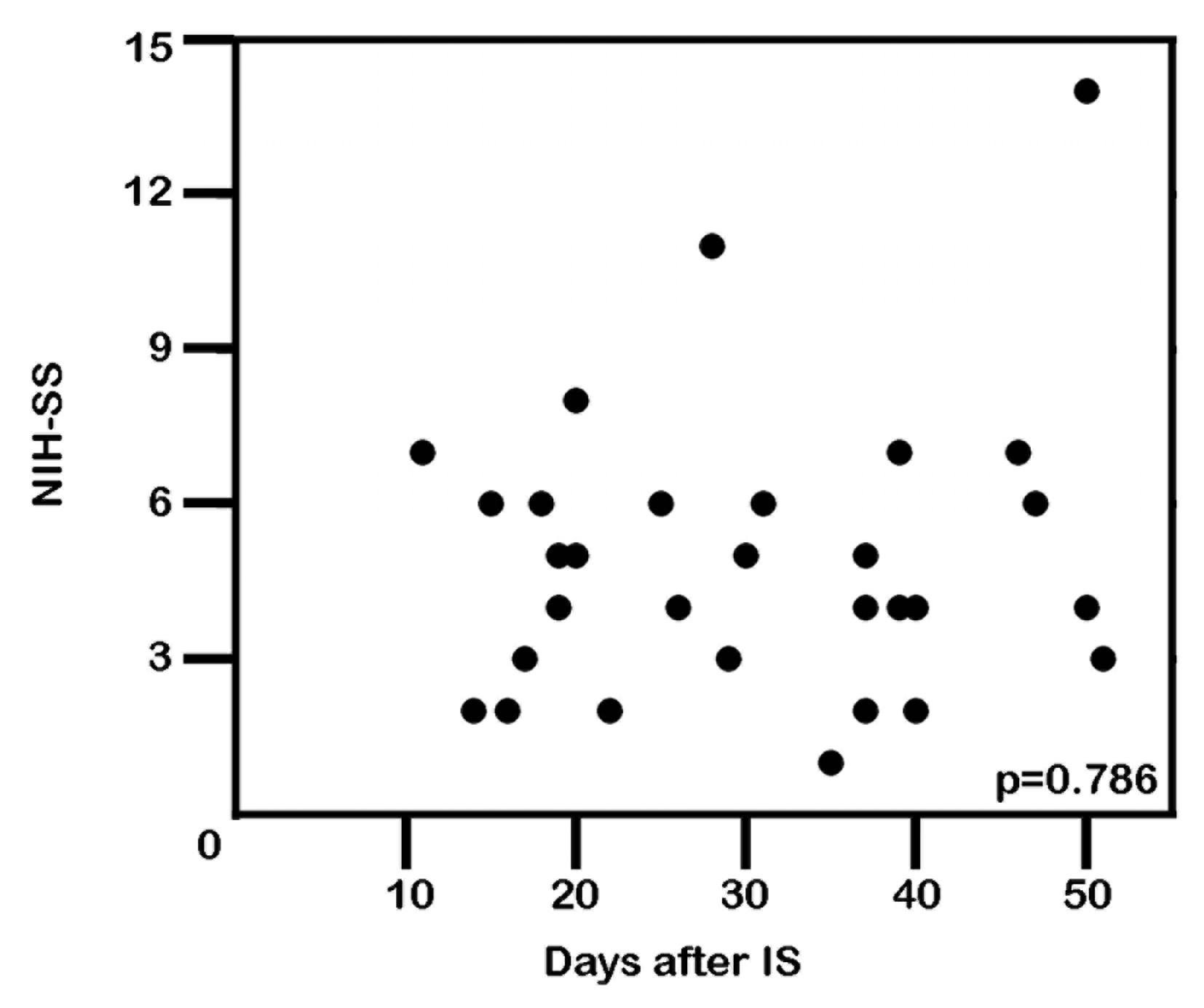


**Fig. S2 Analysis of the correlation of timing of blood collection of IS patients and NIH-SS.** Stoke severity is not related to the time of sample processing in IS patients (ANOVA value p=0.786).

**
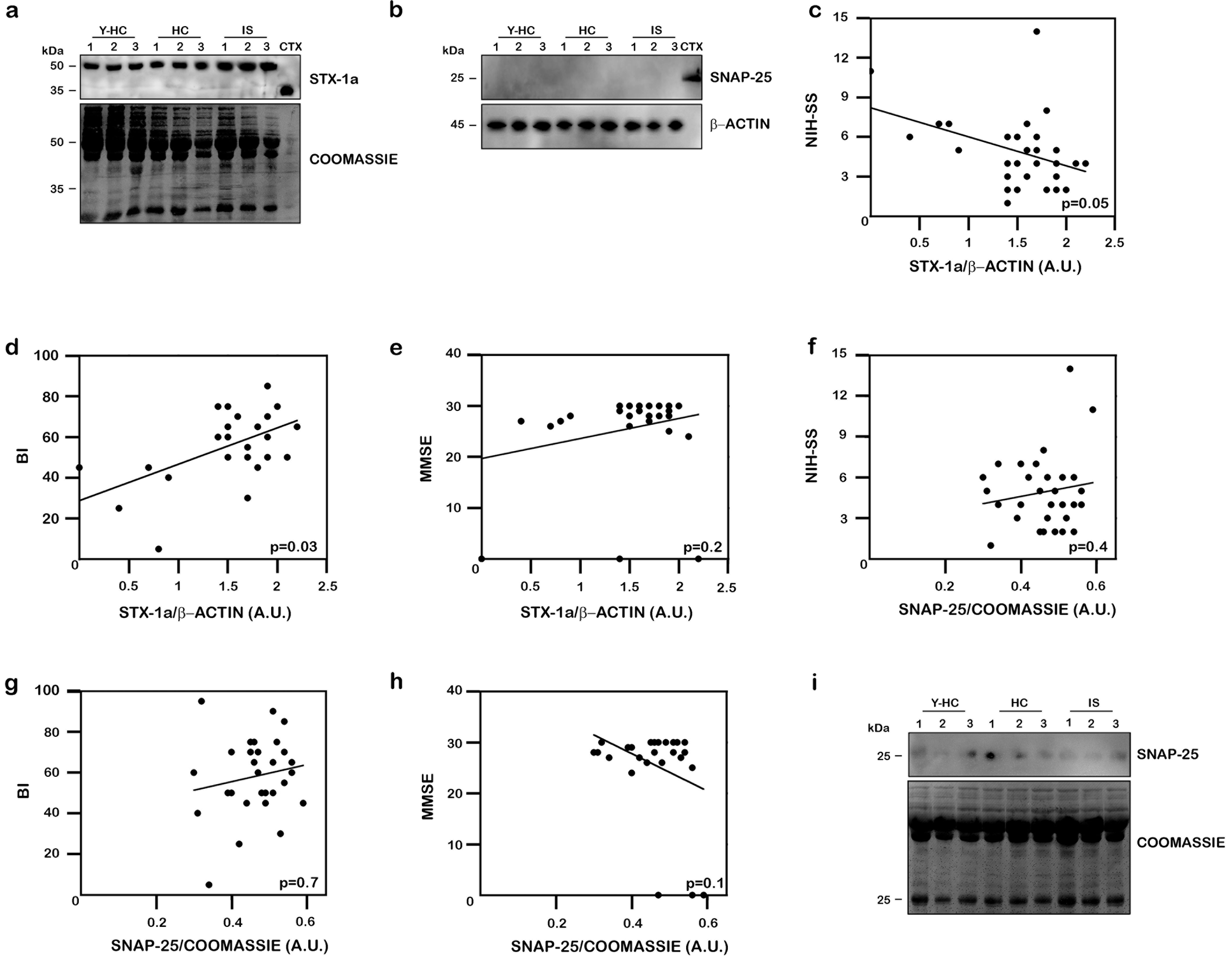
**

**Fig. S3 Expression levels of STX-1a and SNAP-25 in blood of IS patients, HC and Y-HC subjects.** **a) Representative WB analysis of STX-1a in sera of IS patients and healthy controls.** STX-1a is shifted at higher M.W. in all population analyzed. **b) Representative WB analysis of SNAP-25 in PBMCs of IS patients and healthy controls.** SNAP-25 is not expressed in PBMCs in any population analyzed. **c, d) Spearman’s correlation analysis between expression of STX-1a in PBMCs and NIH-SS (c) and BI (d).** STX-1a normalized expression levels in PBMCs of IS patients present a negative association with NIH-SS (**c**) and a positive association with BI (**d**). Both correlations are weak and statistically significant (-0.314, p=0.05 and 0.362, p=0.03 respectively for NIH-SS and BI). **e) Pearson’s correlation analysis between expression of STX-1a in PBMCs and MMSE.** STX-1a normalized expression levels in PBMCs of IS patients present a weak, positive but not statistically significant (p=0.2) correlation with MMSE (0.227). **f, g, h) Pearson’s correlation analysis between expression of SNAP-25 in sera and the three clinical scales: NIH-SS (f), BI (g) and MMSE (f).** SNAP-25 normalized expression levels in sera of IS patients present a very weak positive correlation with NIH-SS (0.154) (**h**) and BI (0.071) (**e**) and a weak negative correlation with MMSE (-0.326) (**f**). All the correlations are not statistically significant (p=0.4, 0.7 and 0.1 respectively for NIH-SS, BI and MMSE). **i) Representative WB analysis of SNAP-25 in the flow through of sera of IS patients and HC subjects depleted from IgG and albumin.** After the stripping of IgG and albumin from sera no specific signal for SNAP-25 is detected in any population analyzed.

For WB analysis 100 µg of sera or PBMC, 0.5 µg of mouse brain cortex lysate and 10 µL of flow through has been loaded in each lane. Uncropped WB and coomassie stainings reported in a, b and g) have been reported in Fig. S12.

**
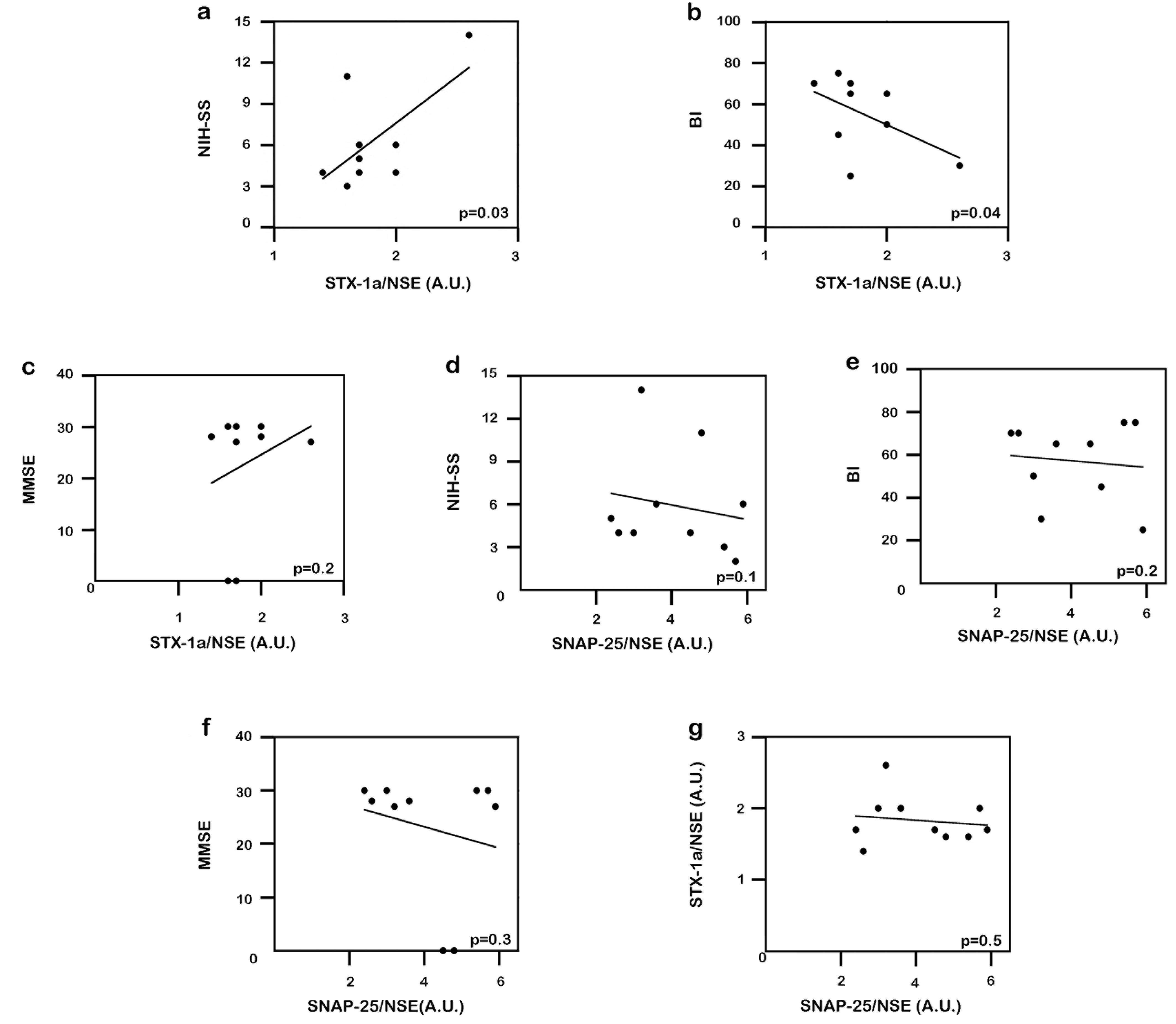
**

**Fig. S4 Spearman’s and Pearson’s correlation analysis between STX-1a and SNAP-25 in NDEs and NIH-SS, BI and MMSE.** a, b) **Spearman’s correlation analysis between expression of STX-1a in NDEs and** **NIH-SS (a) and BI (b).** STX-1a normalized expression levels of IS patients presents a positive correlation with NIH-SS (**a**) and a negative correlation with BI (**b**). Both associations are strong and statistically significant (0.65, p=0.03 for NIH-SS and -0.622, p=0.04 for BI). **c) Pearson’s correlation analysis between expression of STX-1a in NDEs and MMSE.** STX-1a normalized expression levels of IS patients present a weak, positive and not statistically significant (p=0.2) correlation with MMSE (0.255**). d, e, f) Pearson’s correlation analysis between expression of SNAP-25 in NDEs and the three clinical scales: NIH-SS (d), BI (e) and MMSE (f).** SNAP-25 normalized expression levels of IS patients present a very weak negative correlation with NIH-SS (-0.178) (**c**) and BI (-0.111) (**d**) and a weak negative correlation with MMSE (-0.215) (**e**). All the correlations are not statistically significant (p=0.1, 0.2 and 0.3 respectively for NIH-SS, BI and MMSE). **g) Pearson’s correlation analysis between expression of STX-1a and SNAP-25 in NDEs.** STX-1a and SNAP-25 normalized expression levels of IS patients are weak negative correlate (-0.144, p=0.5).

**
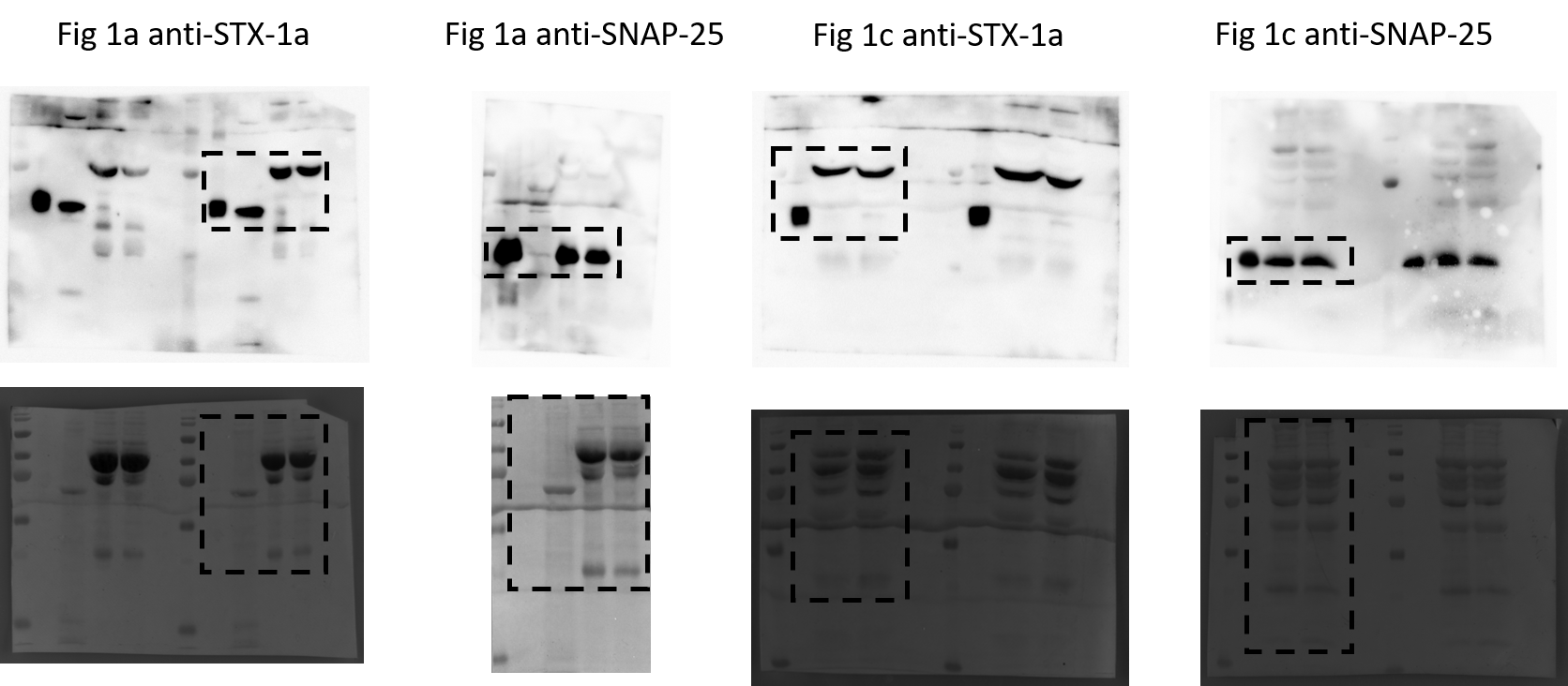
**

**Fig. S5 Original blot and coomassie images reported in Fig. 1.** Cropped blots reported in Fig. 1a and 1c (for both anti-STX-1a, anti-SNAP-25 as well as coomassie staining) have been highlighted with dashed lines in each full-length image.


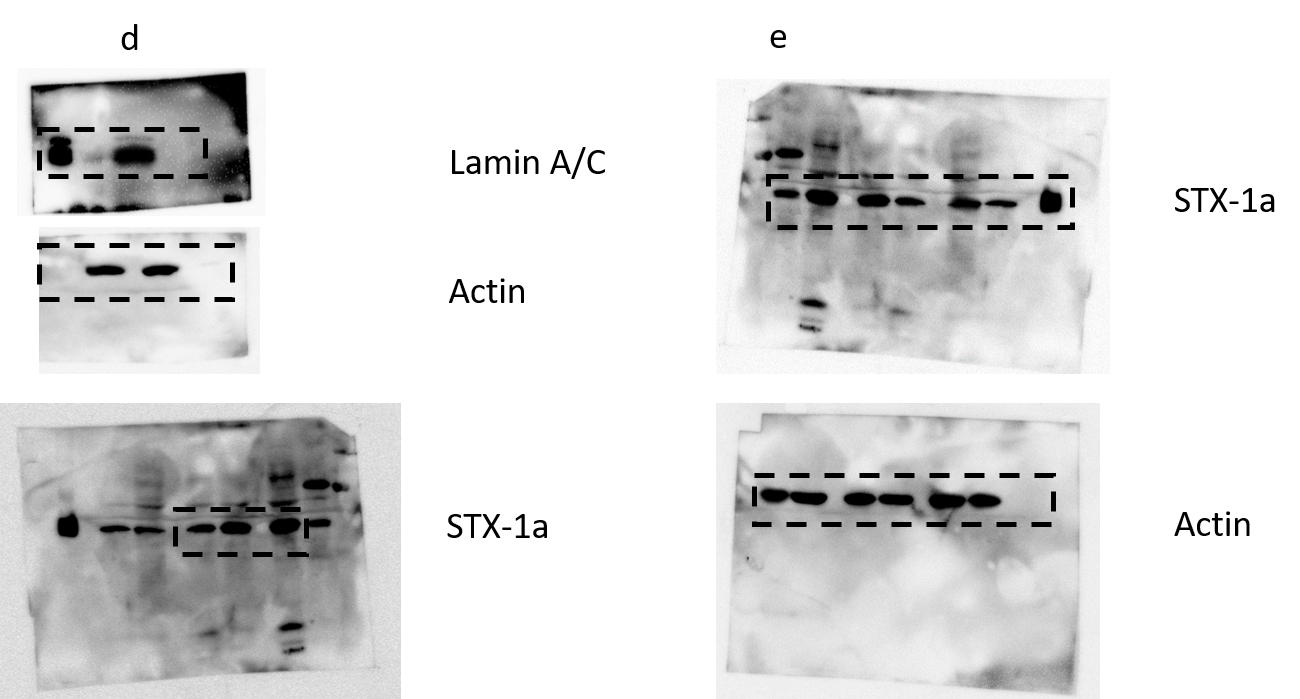


**Fig. S6 Original blot images reported in Fig. 2.** Cropped blots reported in Fig. 2d (for anti-Lamin A/C, anti-β-actin and anti-STX-1a staining) and in Fig. 2e (for anti-STX-1a and anti-β-actin staining) have been highlighted with dashed lines in each full-length image.


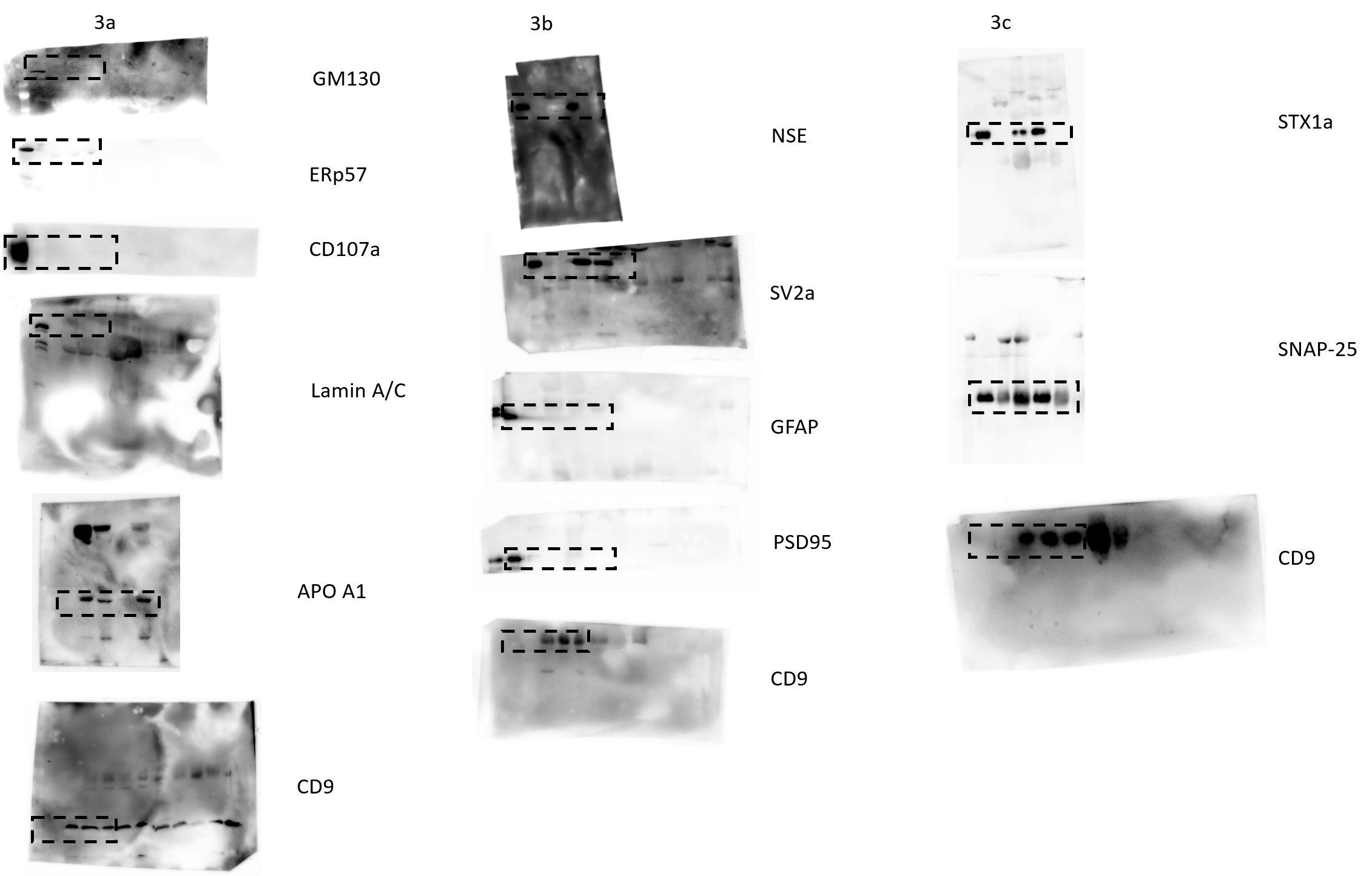


**Fig. S7 Original blot images reported in Fig. 3.** Cropped blots reported in Fig. 3a (for anti-GM130, anti-ERp57, anti-CD107a, anti-Lamin A/C, anti-APO A1 and anti-CD9 staining), in Fig. 3b (for anti-NSE, anti-SV2a, anti-GFAP, anti-PSD95 and anti-CD9) and in Fig. 3c (for anti-STX-1a, anti-SNAP-25 and anti-CD9) have been highlighted with dashed lines in each full-length image.


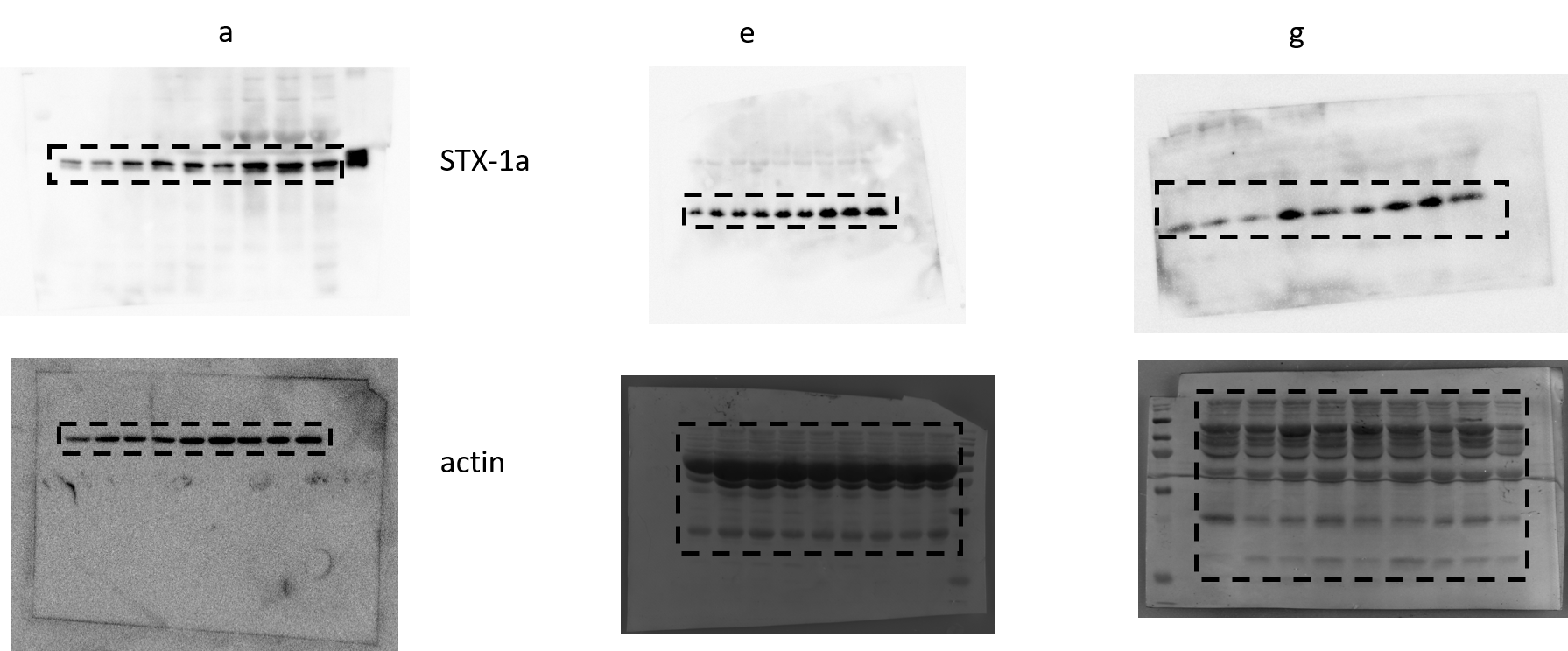


**Fig. S8 Original blot images reported in Fig. 4.** Cropped blots reported in Fig. 4a (for anti-STX-1a and anti-β-actin staining) and in Fig. 4e and 4g (for anti-SNAP-25 as well as coomassie staining) have been highlighted with dashed lines in each full-length image.


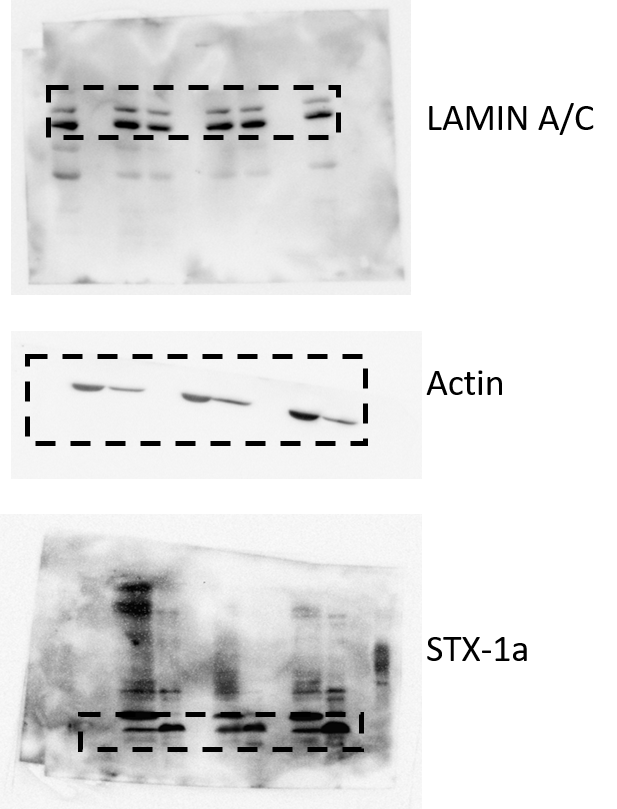


**Fig. S9 Original blot images reported in Fig. 5.** Cropped blots reported in Fig. 5a (for anti-Lamin A/C, anti-β-actin and anti-STX-1a staining) have been highlighted with dashed lines in each full-length image.


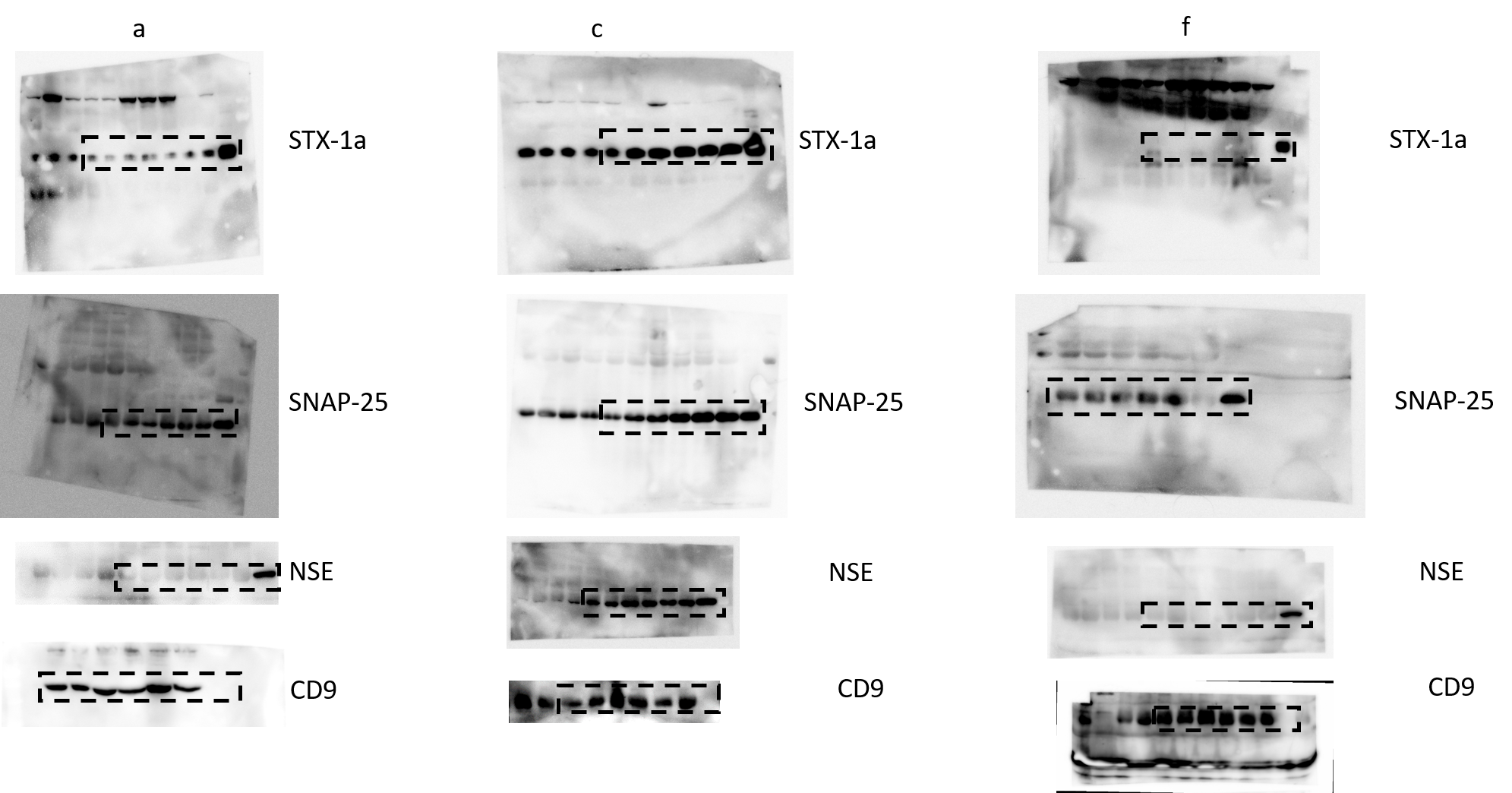


**Fig. S10 Original blot images reported in Fig. 6.** Cropped blots reported in Fig. 6a, c and f (for anti-STX-1a, anti-SNAP-25, anti-NSE and anti-CD9 staining) have been highlighted with dashed lines in each full-length image.


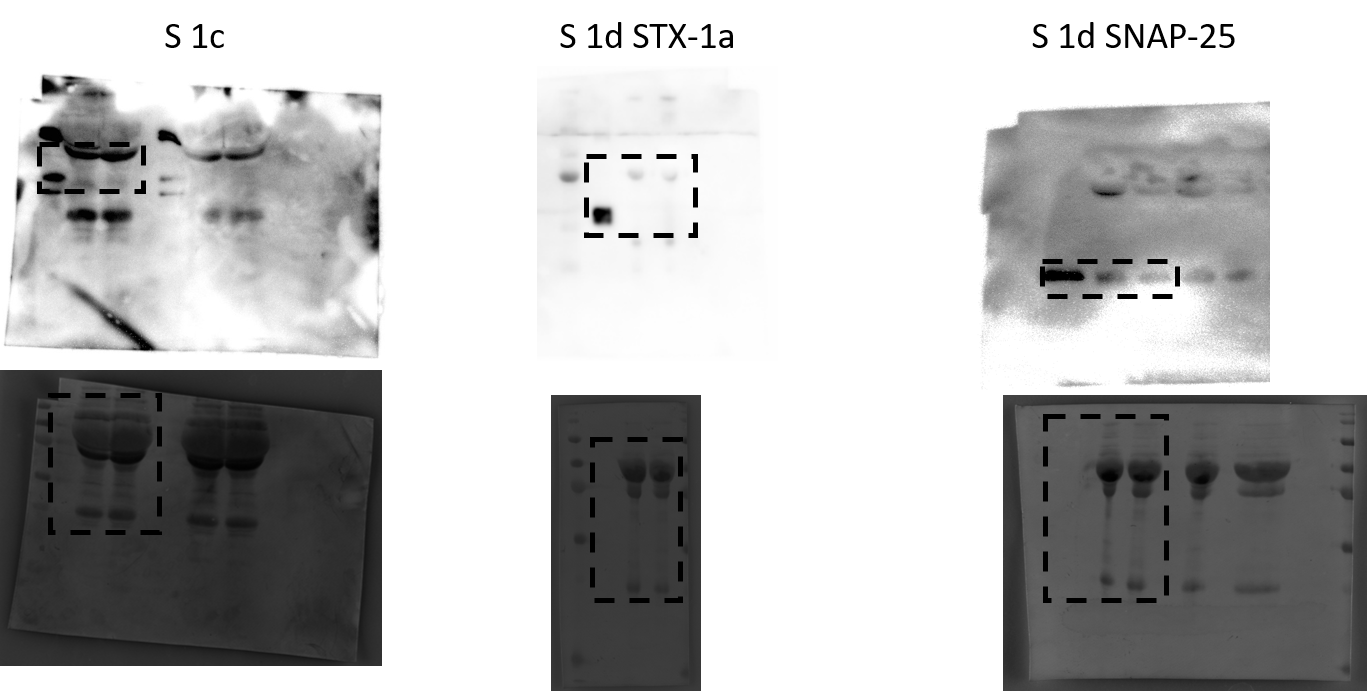


**Fig. S11 Original blot and coomassie images reported in Fig. S2.** Cropped blots reported in Fig. S1c (for anti-STX-1a as well as coomassie staining) and in Fig. S1d (for anti-SNAP-25 as well as coomassie staining) have been highlighted with dashed lines in each full-length image.


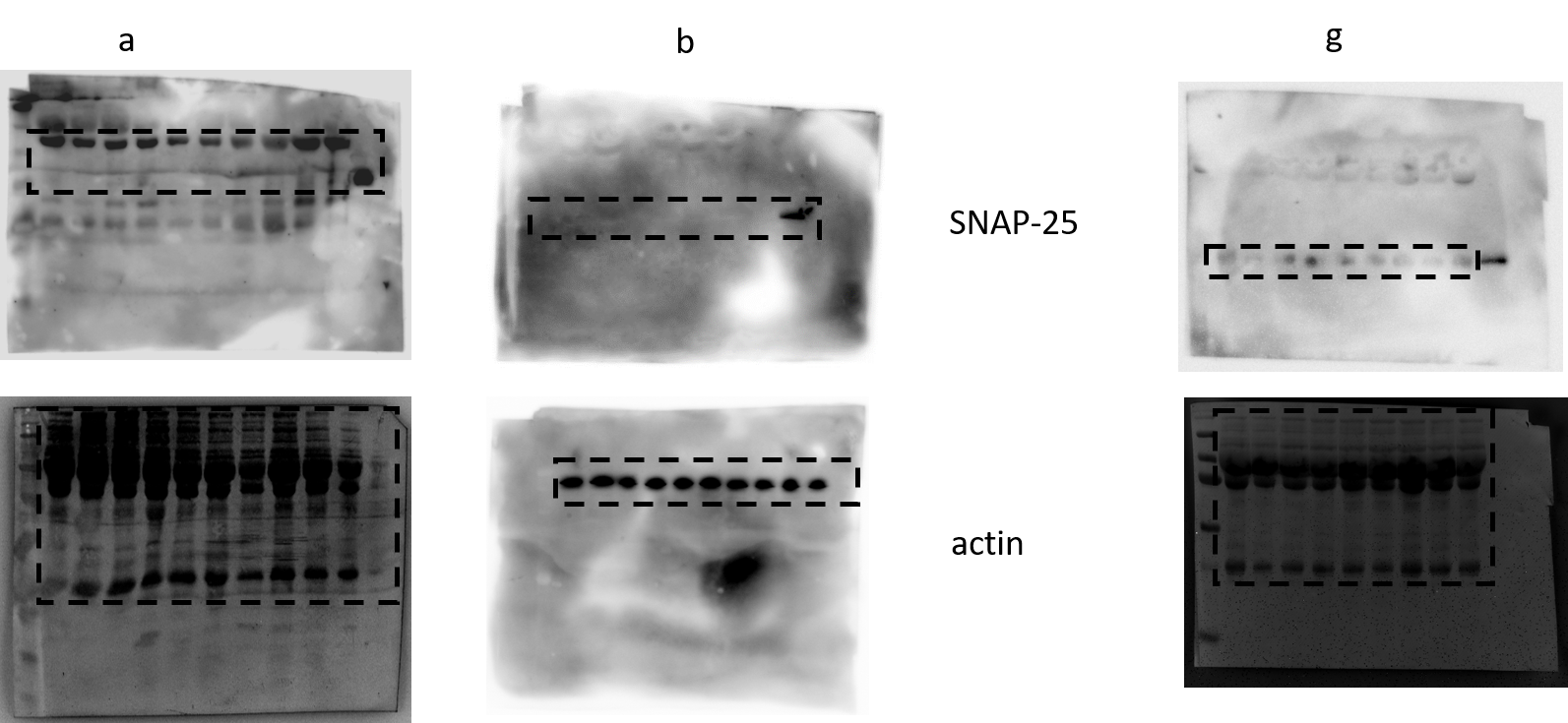


**Fig. S12 Original blot images reported in Fig. S3.** Cropped blots reported in Fig. S2a (for both anti-STX-1a, anti-SNAP-25 as well as coomassie staining), in Fig. S2b (for anti-SNAP-25 and anti-β-actin staining) and in Fig. S2g (for anti-SNAP-25 as well as coomassie staining) have been highlighted with dashed lines in each full-length image.

| **Antibody** | **Brand** | **Experimental procedures** | **Dilutions** | **Figures** | **References** |
| --- | --- | --- | --- | --- | --- |
| APO A1 | Biorbyt | WB | 1:500 | Fig. 3a. | [1] |
| Β-actin (ACTB) | Biorbyt | WB and IF | WB 1:10000  IF 1:50 | Fig. 2a, d, e; Fig. 4a; Fig. S7b; Fig. 5a, c. | - |
| CD9 | Elabsciences | WB | 1:500 | Fig. 3a, b, c; Fig. 6a, c, f. | [2] |
| CD107a | Exbio antibodies | WB | 1:2000 | Fig. 3a. | [3] |
| ERp57 | Elabsciences | WB | 1:2000 | Fig. 3a. | [4] |
| GM130 | Biosciences | WB and IF | WB 1:200  IF 1:100 | Fig. 2a; Fig. 3a. | [5] |
| GFAP | Cell Signaling | WB | 1:1000 | Fig. 3b. | [6] |
| Lamin A/C | Cell Signaling | WB | 1:200 | Fig. 2d, Fig. 3a, Fig. 5a. | [7, 8] |
| NSE | Biorbyt | WB | 1:400 | Fig. 3b; Fig. 6a, c, f. | - |
| PSD95 | Cell Signaling | WB | 1:500 | Fig. 3b. | [9, 10] |
| SNAP-25 | Atlas | WB and IF | WB 1:200  IF 1:200 | Fig. 1a, b, c; Fig. S2a, d, Fig. 3c; Fig. 4e, g, Fig. S7b, g; Fig. 6a, c, f. | [11, 12] |
| STX-1a | AbCam | WB | 1:750 | Fig. S2c; Fig. 3c; Fig. 6a, c, f. | - |
| STX-1a | Elabsciences | WB and IF | WB 1:350  IF 1:200 | Fig. 1a, b, c; Fig. S2a, d; Fig. 2a, d, e; Fig. 4a; Fig. S7a; Fig. 5a, c. | [13] |
| STX-1a | Synaptic System | IF | 1:200 | Fig. S7b. | [14] |
| SV2a | Cell Signaling | WB | 1:200 | Fig. 3b. | [15, 16] |

**Table S1. List of antibodies utilized in this study for both WB and IF experiments.**

**Reference list of antibodies utilized:**

1. Karimi, N. *et al*. Detailed analysis of the plasma extracellular vesicle proteome after separation from lipoproteins. *Cell Mol Life Sci*. **75**(15), 2873–86 (2018). doi:10.1007/s00018-018-2773-4.
2. Yáñez-Mó, M., Tejedor, R., Rousselle, P., & Sánchez –Madrid, F. Tetraspanins in intercellular adhesion of polarized epithelial cells: spatial and functional relationship to integrins and cadherins. *J Cell Sci.* **114**(Pt 3), 577-87 (2001). doi: 10.1242/jcs.114.3.577.
3. Majer, F. *et al*. Danon disease: a focus on processing of the novel LAMP2 mutation and comments on the beneficial use of peripheral white blood cells in the diagnosis of LAMP2 deficiency.*Gene*. **498**(2), 183-95 (2012). doi: 10.1016/j.gene.2012.02.004.
4. Hulpke, S., Baldauf, C. & Tampé, R. Molecular architecture of the MHC I peptide-loading complex: one tapasin molecule is essential and sufficient for antigen processing. *FASEB J*. **26**(12), 5071-80 (2012). doi: 10.1096/fj.12-217489.
5. Ireton, R.C. *et al*. A novel role for p120 catenin in E-cadherin function. *J Cell Biol*. **159**(3), 465-76 (2002). doi: 10.1083/jcb.200205115.
6. Jessen, K.R., Morgan, L., Stewart, H.J. & Mirsky, R. Three markers of adult non-myelin-forming Schwann cells, 217c(Ran-1), A5E3 and GFAP: development and regulation by neuron-Schwann cell interactions. *Development*. **109**(1), 91-103 (1990). doi: 10.1242/dev.109.1.91.
7. Goldberg, M. *et al*. The tail domain of lamin Dm0 binds histones H2A and H2B. *Proc Natl Acad Sci U S A*. **96**(6), 2852-7 (1999). doi: 10.1073/pnas.96.6.2852.
8. Yabuki, M. *et al*. Role of nuclear lamins in nuclear segmentation of human neutrophils. *Physiol Chem Phys Med NMR*. **31**(2), 77-84 (1999).
9. Chetkovich, D.M., *et al*. Postsynaptic targeting of alternative postsynaptic density-95 isoforms by distinct mechanisms. *J Neurosci*. **22**(15), 6415-25 (2002). doi: 10.1523/JNEUROSCI.22-15-06415.2002.
10. Cai, C., Li, H., Rivera, C. & Keinänen, K. Interaction between SAP97 and PSD-95, two Maguk proteins involved in synaptic trafficking of AMPA receptors. *J Biol Chem*. **281**(7), 4267-73 (2006). doi: 10.1074/jbc.M505886200.
11. Chapman, E.R., An, S., Barton, N. & Jahn R. SNAP-25, a t-SNARE which binds to both syntaxin and synaptobrevin via domains that may form coiled coils. *J Biol Chem*. **269**(44), 27427-32 (1994).
12. Greber-Platzer, S., Fleischmann, C., Nussbaumer, C., Cairns, N. & Lubec, G. Increased RNA levels of the 25 kDa synaptosomal associated protein in brain samples of adult patients with Down Syndrome. *Neurosci Lett.* **336**(2), 77-80 (2003). doi: 10.1016/s0304-3940(02)01150-3.
13. Bennett, M.K., Calakos, N. & Scheller R. H. Syntaxin: a synaptic protein implicated in docking of synaptic vesicles at presynaptic active zones. *Science.* **257**(5067), 255-9 (1992). doi: 10.1126/science.1321498.
14. Nagao, M *et al*. Potential Protection Against Type 2 Diabetes in Obesity Through Lower CD36 Expression and Improved Exocytosis in β-Cells. *Diabetes*. **69**(6), 1193-1205 (2020). doi: 10.2337/db19-0944.
15. Janz, R., Goda, Y., Geppert, M., Missler, M. & Südhof, T.C. SV2A and SV2B function as redundant Ca2+ regulators in neurotransmitter release. *Neuron.* **24**(4), 1003-16 (1999). doi: 10.1016/s0896-6273(00)81046-6.
16. van Vliet, E.A., Aronica, E., Redeker, S., Boer, K. & Gorter, J.A. Decreased expression of synaptic vesicle protein 2A, the binding site for levetiracetam, during epileptogenesis and chronic epilepsy. *Epilepsia.* **50**(3), 422-33 (2009). doi: 10.1111/j.1528-1167.2008.01727.x.
